# PCSK9 and coronary atherosclerosis progression beyond LDL‐cholesterol in coronary artery disease patients

**DOI:** 10.1111/eci.70083

**Published:** 2025-06-04

**Authors:** Rosetta Ragusa, Silvia Rocchiccioli, Serena Del Turco, Antonio Morlando, Giuseppina Basta, Arthur Scholte, Danilo Neglia, Chiara Caselli

**Affiliations:** ^1^ CNR, Institute of Clinical Physiology Pisa Italy; ^2^ Department of Cardiology, Heart Lung Center Leiden University Medical Centre Leiden The Netherlands; ^3^ Fondazione Toscana G. Monasterio Pisa Italy

**Keywords:** coronary artery disease, coronary atherosclerosis progression, inflammation, PCSK9

## Abstract

**Background:**

This study evaluated whether plasma PCSK9 is associated with coronary plaque progression in patients with coronary artery disease (CAD) and assessed its involvement in molecular processes of atherogenesis.

**Methods:**

Plasma PCSK9 was measured in 159 patients with stable CAD submitted to coronary computed tomography angiography (CTA) at baseline and after a follow‐up of 6.5 ± 1.1 years. Plaque progression was defined as the annual increase in Total, Fibrous, Fibro‐fatty, Necrotic‐Core and Dense‐Calcium plaque volumes (PV). Pathways linked with PCSK9 were studied by RNA‐sequencing of whole blood and in vitro studies using endothelial cells (EC).

**Results:**

At multivariable analysis, plasma PCSK9 was associated with an annual increase in Necrotic‐Core PV (*p* = .022) independent of cardiovascular risk factors, molecular markers, and medications, including LDL‐C and statins. At RNA‐seq analysis, PCSK9 was linked to the expression of genes involved in the innate‐immune response. Treating EC with PCSK9 resulted in a significant increase in ICAM‐1, VCAM‐1, MCP1 and IL6 mRNA expression.

**Conclusions:**

In patients with CAD, plasma PCSK9 is associated with progression of Necrotic Core‐PV. The link with inflammatory pathways suggested for PCSK9 a potential role for the occurrence of prognostically adverse plaque phenotypes beyond LDL‐C regulation.

## Graphical abstract

In a population of patients with stable CAD, circulating levels of PCSK9 are independently associated with the progression of adverse coronary plaque phenotypes, as assessed by serial coronary computed tomography angiography. At RNA‐seq analysis of whole blood, PCSK9 was linked to the expression of genes involved in the innate‐immune response. Thus, PCSK9 is involved in molecular processes of atherosclerosis possibly conditioning its progression beyond LDL‐Cholesterol.

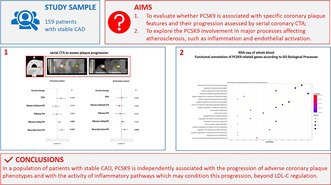

## 1 INTRODUCTION

Cardiovascular diseases account for the largest proportion of deaths and disability in Western countries and among these ischemic heart disease accounts for the largest proportion.^1^, ^2^ Reduction in low‐density lipoprotein cholesterol (LDL‐C), mainly with statins, has decreased the risk of cardiovascular events over the last few decades.^3^ Loss‐of‐function mutations of the proprotein convertase subtilisin/kexin type 9 (PCSK9), a protein involved in cholesterol homeostasis by enhancing degradation of the hepatic low‐density lipoprotein receptor (LDLR),^4^ are associated with low plasma LDL‐C levels and decreased risk of cardiovascular events.^5^ Clinical studies have demonstrated that inhibition of PCSK9 potently reduces serum LDL‐C concentrations by 50%–70% when administered both as a monotherapy and on a background therapy with statins.^6^

Recent pre‐clinical studies have shown a key role of PCSK9 in the initiation and progression of atherosclerosis by mechanisms independent of LDLR regulation,^7^, ^8^, ^9^ including potential PCSK9 effects on relevant inflammatory pathways.^10^, ^11^ However, despite the pre‐clinical evidence, to date, data on the association between PCSK9 and human coronary disease progression are lacking.

New coronary imaging approaches can be employed to assess coronary plaque burden, coronary artery lumen dimensions and plaque features.^12^ The natural course of atherosclerosis typically involves progression, which can be complicated by adverse events such as plaque rupture or erosion. Different clinical presentations are associated with different stages of plaque development, including asymptomatic disease (characterized by intimal thickening and thick cap fibroatheroma), unstable lesions (characterized by thin fibrous cap atheroma, calcified nodules, necrotic core and inflammatory infiltrates) which may lead to myocardial infarction, and stable obstructive lesions (fibrocalcific plaque) which may cause stable angina.^13^, ^14^ Consequently, monitoring plaque features helps to guide the optimization of lipid‐lowering and disease‐modifying treatments and is of utmost importance in evaluating and reducing coronary atherosclerotic risk.^14^ Coronary computed tomography angiography (CTA) is a well‐established non‐invasive imaging tool with high diagnostic performance for recognizing and characterizing coronary atherosclerosis and has a high predictive value for adverse CV events.^15^

The purpose of the present study is to evaluate whether PCSK9 is associated with specific coronary plaque features and their progression assessed by serial coronary CTA in a population of patients with chronic CAD. Moreover, it explores the possible involvement of PCSK9 in the major processes affecting vascular atherosclerosis, such as inflammation and endothelial activation. Specifically, a gene expression study was performed by RNA sequencing using whole blood samples from CAD patients in order to identify the processes associated with PCSK9 expression, and an in vitro study to assess the effect of PCSK9 on endothelial activation.

## 2 MATERIALS AND METHODS

### 2.1 Study design and population

The “Simulation Modeling of coronary ARTery disease: a Tool for clinical decision support” (SMARTool) study was a prospective, international, multicenter observational study in which patients who previously underwent coronary CTA for suspected CAD were prospectively included to undergo clinical, molecular and coronary CTA follow‐up. The list of eligibility, inclusion, exclusion and exit criteria of SMARTool Clinical Study were previously reported in detail.^16^ Blood samples were collected from patients in a fasting state during the clinical visit before coronary CTA, and aliquots were stored at IFC Biobank. Among the 212 patients whose CTA scan were evaluated by quantitative analysis and with available biological samples, 186 were selected to be included in the HURRICANE study. Among them, 159 patients with available information on clinical and molecular profiles, and on gene expression analysis of whole blood samples at follow up, were included in the present study. The study flow chart is reported in Figure S1.

The protocol of the SMARTool and HURRICANE studies was approved by local ethics committees and conformed to the ethical guidelines of the 1975 Declaration of Helsinki; all patients gave their written informed consent to participate, and the procedures followed were in accordance with institutional guidelines.

### 2.2 Clinical features, PCSK9 measurements and molecular profile

Information on cardiovascular risk factors, including age, sex, family history of CAD, smoking status, diabetes mellitus, hypertension, obesity, medication use and molecular profiles, were collected.^17^ PCSK9 plasma levels were measured in blood samples stored in the SMARTool biobank by a dedicated ELISA (Quantikine ELISA, R&D Systems) (.07 ± .07 ng/L LoD; 3.96% intra‐run variation; 8.33% inter‐run variation). Additional specific metabolic and inflammatory biomarkers were measured at baseline and at follow‐up in all patients using standard methods. LDL cholesterol was calculated according to the Friedewald formula.

### 2.3 Coronary CTA Analysis

The methodology for coronary CTA imaging acquisition and analysis has been previously reported in detail.^16^ First, a visual, side‐by‐side analysis of the baseline and follow‐up coronary CTAs was performed to assess the presence and severity of coronary plaques. Obstructive CAD was defined as the presence of lumen stenosis >50% in all segments.

Subsequently, quantitative CTA analysis was performed for all visually determined plaques, using a dedicated software package (QAngio CT Research Edition version 3.1.2.0). Baseline and follow‐up coronary lesions were matched using fiduciary landmarks (e.g. side branches, distance from the ostium) and analysed side by side. The complete workflow of quantitative CTA analysis has been described in detail previously.^16^ For each coronary lesion, total plaque volume (TPV) and plaque volume (PV) according to the plaque composition (Fibrous, Fibrous‐Fatty, Necrotic Core and Dense Calcium) were determined.

CAD progression was defined as the absolute increase in plaque volume by quantitative CTA analysis on a per patient basis. Per‐patient plaque volume was calculated by summing the plaque volumes of individual coronary plaques. Total, Necrotic Core, Fibro‐fatty, Fibrous plaque and Dense Calcium PV progression were assessed on a per‐patient basis and adjusted for the time interval between the baseline and follow‐up coronary CTA (i.e., the interscan period). Accordingly, the annual PV change was calculated as follows: (PV at follow‐up − PV at baseline)/(interscan period).

### 2.4 RNA extraction from Whole blood, sequencing and analysis

Total RNA was extracted from whole blood samples from patients at follow up using MagMax for stabilized Blood Tubes RNA isolation kit (Thermofisher). mRNA profiling analysis was carried out providing sequencing of blood. mRNA Sequencing libraries were prepared from 500 ng of total RNA. First, mRNA selection was performed using oligo(dT) beads. Then mRNA was converted into sequencing library according to the IlluminaTruSeq stranded mRNA protocol. Sequencing was performed on the HiSeq2500 platform, using paired‐end sequencing (2 × 50 bp). Raw data were aligned to the human reference genome hg19/GRCh37 and gene expression levels quantified using STAR and gene models of the Ensembl release 75. Gene expression values were calculated as RPKM (reads per kilobase per million mapped reads) and median‐normalized across the cohort to ensure comparability between samples.

### 2.5 Bioinformatic analysis

In the study of gene expression, the expression of genes as emerged by RNA‐seq of whole blood was used for a correlation analysis to determine the strength and statistical significance of their association with PCSK9 in peripheral circulation and with PCSK9 mRNA expression in whole blood. Firstly, PCSK9 plasma levels were correlated with the expression of genes as emerged by the RNA sequencing of whole blood. The analysis produced a correlation coefficient (*r*) between PCSK9 and each gene, with statistical significance evaluated using *p* values and correlations were deemed statistically significant when *p* < .05. Molecular pathway enrichment was performed via STRING (Version 12.0) to link gene datasets with higher order functional information, as described by the Kyoto Encyclopedia of Genes and Genomes (KEGG) databases. Next, a correlation analysis was employed to determine the association between PCSK9 mRNA expression and the expression of other genes involved in the “Lipid and Atherosclerosis” pathway, as defined by the KEGG (hsa05417).^18^ The analysis produced a correlation coefficient (*r*) for each gene pair, with statistical significance evaluated using *p* values. Correlations were deemed statistically significant when *p* < .001. To analyse the data, mRNA count matrices were processed using R software (version 4.3.0). Genes showing a significant correlation with PCSK9 were subsequently characterized by Gene Ontology (GO) over‐representation analysis, which focused on GO Biological Processes (BPs). This analysis was conducted using the clusterProfiler package (version 4.8.3) and the OrgDb annotation database (Genome‐wide annotation for Human, version 3.17.0), providing insight into the functional roles of the correlated genes. Significant GO terms were identified based on a threshold of *p* < .01, with correction for multiple testing via the False Discovery Rate (FDR) using the Benjamini–Hochberg method.^19^ The top 20 most significant terms from the functional enrichment analysis were visualized using dot plots, which illustrated the enrichment of biological processes in relation to the correlated genes. These visualizations provided an integrated view of the gene sets and their associated biological functions, offering further insights into their roles within lipid metabolism and atherosclerosis.

### 2.6 Cell culture and treatment protocol

Experiments were performed in human umbilical vein endothelial cells (HUVEC) maintained in a specific culture medium following the manufacturer's instructions (Promega, Heidelberg, Germany). Experiments were conducted on confluent cells between passages 2 and 3. HUVEC were stimulated with .25, 1, 2.5 or 5 μg/mL human PCSK9 Protein (ACRObiosystem, Newark, DE, USA) for 4 h to evaluate mRNA expression of VCAM‐1, ICAM‐1, MCP‐1, IL‐6, IL‐8 and for 24 h to assess the protein surface exposure of VCAM‐1 and ICAM‐1.

### 2.7 RNA extraction, cDNA synthesis, and real time PCR

Total RNA was extracted from 10^6^ endothelial cells by the acid guanidinium thiocyanate‐phenol‐chloroform method, and RNA concentration was evaluated spectrophotometrically. First‐strand cDNA was synthesized starting from about 1 μg total RNA as template using iSCRIPT kit (Biorad).

Real‐time PCR reactions were performed in duplicate by the Bio‐Rad C1000 thermal cycler (CFX‐96 real‐time PCR detection systems; Bio‐Rad). In order to monitor cDNA amplification, Eva‐Green (SsoFAST EvaGreen Supermix, Biorad) was used as a fluorophore. The cycling condition included an initial denaturation‐activation step at 98°C for 30 s, followed by 42 cycles at 95°C for 5 s and 60°C for 30 s. A list of primer pairs used for real‐time analysis is reported in Table S1.

### 2.8 Surface enzyme immunoassay (EIA)

After PCSK9 treatment, endothelial cell monolayers were incubated with a mouse anti‐human monoclonal ICAM‐1 IgG (Ab HU5/3) and VCAM‐1 IgG (Ab HU5/3) (undiluted supernatant from the hybridoma cells, American Type Culture Collection, ATCC, Promochem Milan, Italy) followed by peroxidase anti‐mouse IgG (Amersham Life Sciences). The surface exposure of ICAM‐1 and VCAM‐1 was quantified spectrophotometrically (optical density at 450 nm wavelength).

### 2.9 Statistical analysis

In the clinical study, categorical variables are presented as numbers (percentage), continuous variables as mean ± standard deviation (SD). No normally distributed data after Shapiro–Wilk test were Ln transformed. The comparison between clinical, molecular and coronary CTA data, obtained at the time of the first scan and of the second scan, was performed by the paired t‐test or McNemar test as appropriate. Patients were divided into groups according to PCSK9 Tertiles at baseline (Table S2) and Total, Dense Calcium, Fibrous, Fibro‐fatty, and Necrotic Core PV at baseline and follow‐up, and annual change of PV, were compared across PCSK9 Tertiles using Kruskal–Wallis or chi‐square test as appropriate. Univariate and multivariate linear regression were used to estimate the association between baseline PCSK9 and the annual increase in plaque volumes. Models were developed starting from the univariate association and then, to account for possible confounding effects, adjusting for clinical variables including age, sex, risk factors, anti‐ischemic (beta blockers and calcium antagonists), anti‐hypertensive (ACE inhibitors, ARBs, diuretics) and antiplatelets, statins, LDL‐C, IL6, MMP9 and ICAM1. A two‐sided *p*‐value of *p* < .05 was considered statistically significant.

In the in vitro study, two‐group comparisons were performed by the unpaired Student's *t*‐test and multiple comparisons by one‐way ANOVA followed by Fisher's LSD test. Values of *p* < .05 were considered statistically significant.

## 3 RESULTS

### 3.1 Patient characteristics at baseline and follow‐up

Clinical features, molecular profiles and CTA coronary plaque patterns at the time of the first and second CTA scan (interscan period 6.4 ± 1.2 years) are summarized in Table 1. Study participants (*n* = 159) were aged 63 ± 7 at baseline (69 ± 8 years at follow up), 64% were men, and a family history of CAD was present in 44% of the study population. Among risk factors, the frequency of diabetes and hypertension significantly increased from baseline to follow‐up, while the frequency of smoking significantly decreased. Among medications, the use of calcium antagonists, ACE inhibitors, anti‐diabetics and statins significantly increased. As to the molecular profile, while Total‐C and LDL‐C significantly decreased from baseline to follow‐up, there was a significant increase in plasma HDL‐C, TG, IL6, VCAM1 and PCSK9. At CTA scans, a significant increase in TPV, necrotic core and dense calcium PV was observed from baseline to follow‐up, while fibrous PV significantly decreased.

**TABLE 1:** Clinical, molecular and coronary CTA data of the study population at Baseline and Follow‐up.

	Baseline *n* = 159	Follow‐up *n* = 159	*p* Value
*Clinical features*			
*D*emographic			
Age, years	63 ± 7	69 ± 8	**<.001**
Male sex	102 (64)	102 (64)	‐‐‐
Risk factors			
Family history	70 (44)	70 (44)	‐‐‐
Diabetes	37 (23)	46 (29)	.**022**
Hypertension	106 (67)	122 (77)	.**005**
Smoking	26 (16)	17 (11)	.**035**
Obesity	32 (20)	41 (26)	.136
Medications			
Beta‐blockers	75 (47)	83 (52)	.215
Calcium antagonists	18 (11)	35 (22)	.**003**
ACE inhibitors	58 (36)	66 (42)	.302
ARBs	23 (14)	30 (19)	.248
Diuretics	30 (19)	31 (19)	1.000
Anti‐diabetic	26 (16)	40 (25)	.**001**
Statins	82 (52)	113 (71)	**<.001**
Anti‐platelets	108 (68)	100 (63)	.374
*Molecular profile*			
Total‐C, mg/dL	184 ± 49	177 ± 46	.**038**
LDL‐C, mg/dL	110 ± 42	94 ± 41	**<.001**
HDL‐C, mg/dL	51 ± 15	55 ± 15	**<.001**
Triglycerides, mg/dL	120 ± 61	144 ± 99	.**001**
FPG, mg/dL	109 ± 27	108 ± 28	.748
Insulin, IU/	11.7 ± 12.1	12.7 ± 13.4	.683
hs‐CRP, mg/dL	.37 ± .55	.30 ± .39	.116
Interleukin 6, pg/mL	1.09 ± 1.23	2.00 ± 4.36	**<.001**
MMP9, ng/mL	129 ± 186	93 ± 87	.437
ICAM1, ng/mL	198 ± 74	205 ± 83	.573
VCAM1, ng/mL	537 ± 150	614 ± 167	**<.001**
PCSK9, ng/mL	220 ± 126	249 ± 86	**<.001**
*Coronary PVs*			
Total PV, mm^3^	648 ± 595	727 ± 663	**<.001**
Fibrous PV, mm^3^	268 ± 264	248 ± 236	.**039**
Fibrous‐fatty PV, mm^3^	137 ± 130	133 ± 122	.153
Necrotic Core PV, mm^3^	166 ± 158	197 ± 178	**<.001**
Dense Calcium PV, mm^3^	60 ± 94	116 ± 143	**<.001**

*Note*: Continuous variables are presented as mean ± standard deviation, categorical variables as absolute N and (%). Bold *p values* <.05 show statistically significant differences.

### 3.2 Plasma PCSK9 and molecular profiles

**FIGURE 1:**
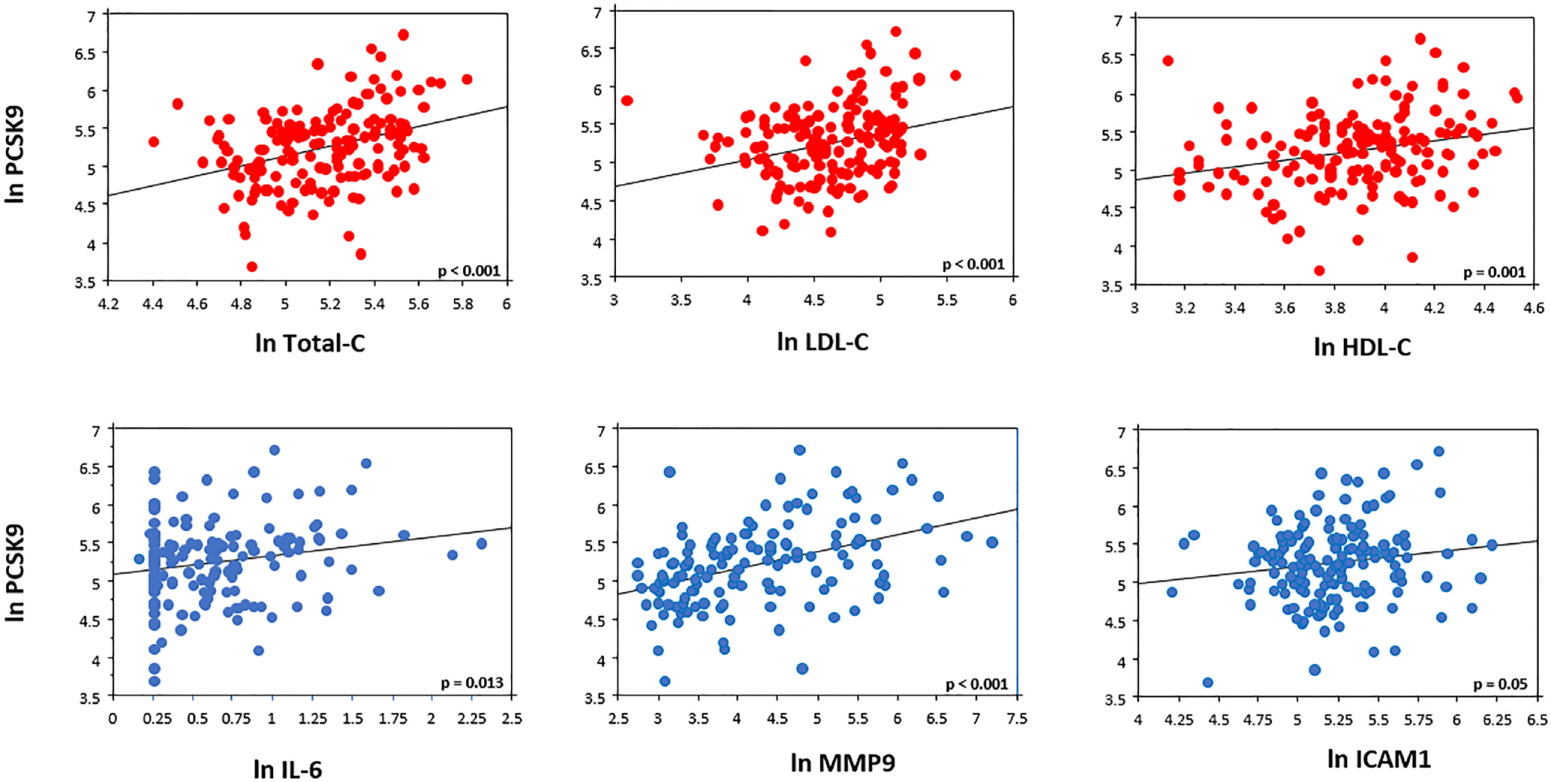
Association between circulating levels of plasma PCSK9 with lipid (Total‐C, LDL‐C and HDL‐C) and inflammatory (IL6, MMP9 and ICAM1) profiles of study patients.

The comparison of clinical features and molecular measurements across plasma PCSK9 tertiles at baseline is reported in Table S2. Male sex and most of the established risk factors were similar across PCSK9 tertiles. Patients in the higher tertiles were more obese, older, more frequently used ACE inhibitors and had higher levels of cholesterol (Total‐C, LDL‐C, HDL‐C) as well as of inflammatory markers (IL6 and MMP9). In the overall population, the PCSK9 plasma levels were positively related to cholesterol, IL6, MMP9 and ICAM1 levels (Figure 1).

### 3.3 Plasma PCSK9 and coronary plaque characteristics and progression

**FIGURE 2:**
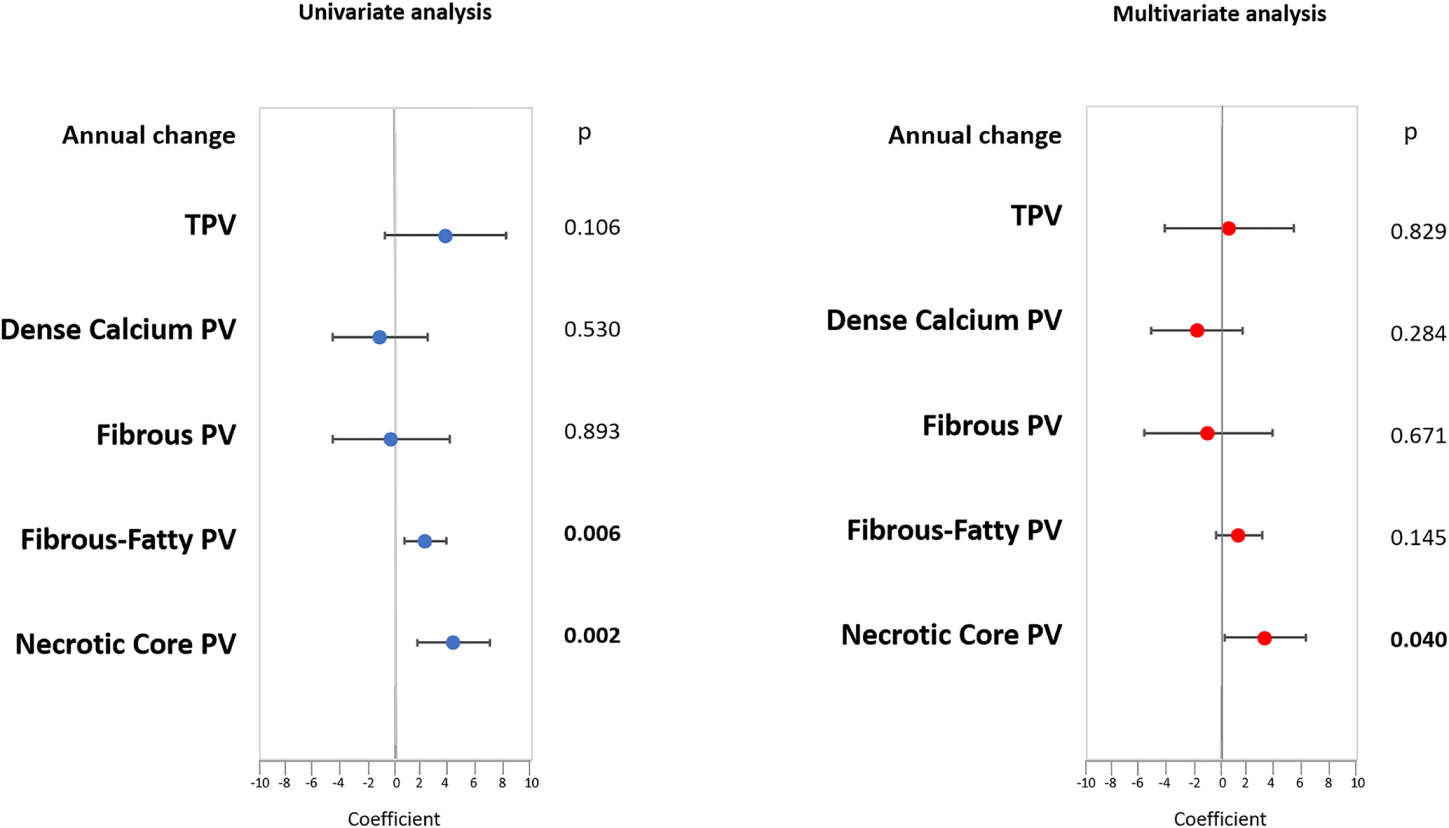
Association of PCSK9 plasma levels and plaque progression. In univariate and multivariate models, data are depicted as β coefficients with 95% CIs for the annual change in plaque volume from baseline to follow‐up coronary CTA.

The comparison of coronary plaque features is reported in patient subgroups defined according to baseline PCSK9 tertiles in Table 2. Total PV, dense calcium, fibrous, fibrous‐fatty and necrotic core PVs at baseline and follow‐up did not differ among baseline PCSK9 tertiles. On the other hand, annual changes of fibrous fatty and necrotic core PVs showed a significant trend to increase among baseline PCSK9 tertiles. At univariate analysis, baseline PCSK9 plasma levels were significantly associated with annual change of fibrous‐fatty PV and necrotic core PV (Figure 2). When the study population was stratified by statin use and sex, the association of PCSK9 levels with annual change of necrotic core PV remained significant in patients with and without statin therapy (Table S3) and in men (Table S4). At multivariable linear regression analysis, after adjustment for baseline confounding factors, the association between PCSK9 plasma levels and necrotic core PV annual change remained significant (Figure 2).

**TABLE 2:** Baseline, follow‐up and annual changes in plaque phenotypes at coronary CTA according to PCSK9 tertiles.

	Tertile I < 153 ng/mL *n* = 53	Tertile II 153–234 ng/mL *n* = 53	Tertile III > 234 ng/mL *n* = 53	*p* Value
Baseline Plaque Volume				
Total PV, mm^3^	607 ± 552	628 ± 534	708 ± 690	.846
Dense Calcium PV, mm^3^	45 ± 60	49 ± 66	85 ± 133	.212
Fibrous PV, mm^3^	237 ± 214	262 ± 251	303 ± 316	.723
Fibrous‐Fatty PV, mm^3^	136 ± 130	135 ± 123	139 ± 140	.979
Necrotic Core PV, mm^3^	173 ± 170	164 ± 142	162 ± 164	.918
Follow up Plaque Volume				
Total PV, mm^3^	671 ± 632	702 ± 612	807 ± 742	.673
Dense Calcium PV, mm^3^	109 ± 130	88 ± 79	151 ± 193	.494
Fibrous PV, mm^3^	222 ± 227	246 ± 227	277 ± 254	.416
Fibrous‐Fatty PV, mm^3^	124 ± 114	132 ± 123	143 ± 131	.764
Necrotic Core PV, mm^3^	183 ± 163	200 ± 177	209 ± 195	.834
Annual change Plaque Volume				
Total PV, mm^3^/year	9.72 ± 14.91	10.87 ± 13.72	16.28 ± 19.29	.165
Dense Calcium PV, mm^3^/year	9.83 ± 12.44	6.3 ± 9.7	10.67 ± 14.85	.663
Fibrous PV, mm^3^/year	−2.47 ± 11.35	−2.65 ± 15.77	−3.75 ± 18.83	.611
Fibrous‐Fatty PV, mm^3^/year	−1.81 ± 4.77	−.93 ± 6.77	.51 ± 5.49	.**014**
Necrotic Core PV, mm^3^/year	1.45 ± 6.55	5.28 ± 11.26	7.55 ± 10.64	.**002**

*Note*: Continuous variables are presented as mean ± standard deviation, categorical variables as absolute N and (%). Bold *p* values show statistically significant differences.

### 3.4 Gene expression study: PCSK9, inflammation, and atherosclerosis progression

**FIGURE 3:**
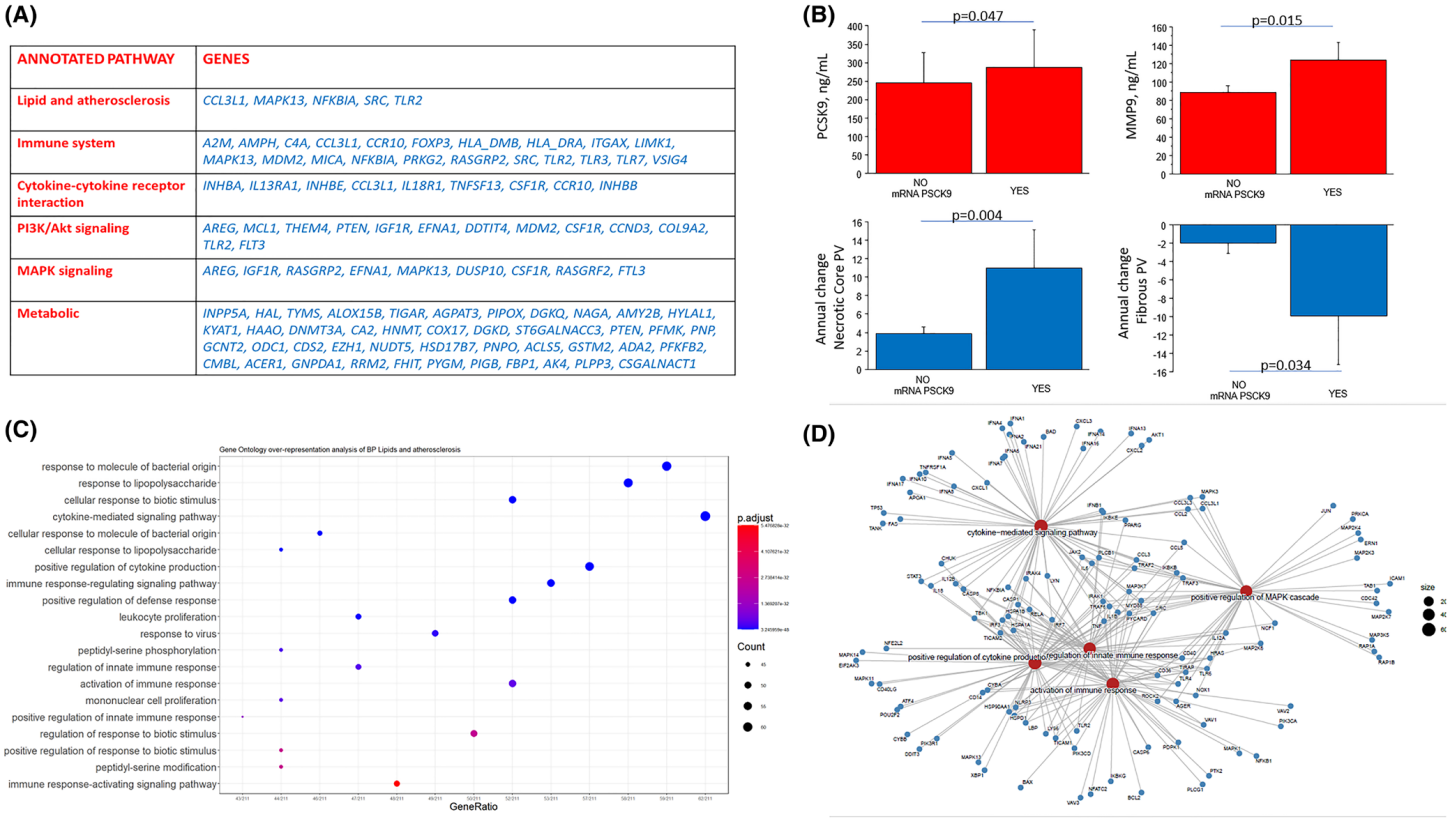
RNA‐sequencing results (A) Molecular pathway enrichment by STRING linking circulating PCSK9‐related gene dataset with KEGG databases; (B) PCSK9 and MMP9 plasma levels together with annual change of Necrotic Core and Fibrous PVs divided according to presence of PCSK9 gene expression in whole blood; (C) Functional annotation of PCSK9‐related genes according to GO Biological Processes. The 20 most significant biological processes are shown. The GeneRatio indicates how many PCSK9‐related genes included in the analysis were annotated to the specific GO biological process. GO terms were filtered for adjusted *p*‐value <.01; (D) Visual combination of genes with related biological processes for enhanced graphical representation of functional categories related to innate immunity.

In order to explore the potential molecular mechanisms associated with PCSK9 modifications in patients with CAD, PCSK9 plasma levels were correlated with gene expression profiles identified through RNA sequencing of whole blood. Among the 538 genes significantly correlating with plasma PCSK9 (Table S5), KLRF2 (*r* = .25), IL18R1 (*r* = .22), TLR2 (*r* = .22), MAPK13 (*r* = .21) are involved in immune response and cytokine regulation; SHISAH3 (*r* = −.24), SRC (*r* = −.23) in foam cell formation; FLT3 (*r* = .31), FKBP5 (*r* = .026), DDIT4 (*r* = .25) in Akt signalling; and DAAM2 (*r* = .32), ADAM11 (*r* = .26), MGP (*r* = .25), NRROS (*r* = −.22), HTRA1 (*r* = .21) and COL9A2 (*r* = .21) in the regulation of extracellular matrix. Plasma PCSK9‐correlated genes were found to belong to lipid and atherosclerosis, immune system, cytokine‐cytokine receptor interaction, PI3K/Akt signalling, MAPK signalling and metabolic pathways belonging to the annotated KEGG pathway databases (Figure 3A).

Moreover, at RNA seq, 13% of study patients expressed *PCSK9* in whole blood at follow‐up. Clinical, molecular and CTA data of the study samples divided according to presence of PCSK9 gene expression in whole blood at follow‐up are reported in Table S6. Circulating levels of PCSK9 and MMP9 were higher in patients expressing PCSK9, which also showed a significant increase in necrotic core PV and a decrease in fibrous PV during follow up (Figure 3B). Among the genes known to be involved in the atherosclerotic process (as reported in the “lipid and atherosclerosis” KEGG pathway), PCSK9 expression showed a significant correlation (*p* < .001) with 157 genes (Table S7). The most PCSK9‐correlated gene were MMP9 (*r* = .68), together with molecules involved in the upstream signalling such as MAPK pathway (MAP2K6, *r* = .59; MAP2K3, *r* = .51; MAPK10, *r* = .40; MAPK3, *r* = .36; MAPK14, *r* = .34). Genes involved in the Toll‐like receptor signalling pathway (TLR2, *r* = .47; LY96, *r* = .44; MYD88, *r* = .19; IRAK1, *r* = .13), inflammasome complex, including NLRP3 (*r* = .40), CASP1 (*r* = .12), and IL‐18 (*r* = .30), and neutrophil cytosolic factor involved in leukocyte migration and neutrophil extracellular trap formation including (NCF1; *r* = .46; NCF4, *r* = .44; NCF2, *r* = .33) were significantly associated with PCSK9 gene expression. Furthermore, regulators of cholesterol metabolism (ApoB, *r* = .34; ApoA1, *r* = .33; ABCG1, *r* = .27) were significantly related to PCSK9 gene expression. Different molecular mediators including PKI3/Akt, CALM, HSPs and PBLC signalling pathways, affecting a variety of cellular activities such as proliferation and apoptosis, were also correlated with PCSK9 expression. Based on GO functional enrichment analysis, the genes associated with PCSK9 expression were functionally characterized using the biological processes ontology and the 20 most significant terms are presented in Figure 3C. PCSK9‐correlated genes were found to be involved in biological processes related with cytokine‐mediated signal pathways and regulation of cytokine production, regulation and activation of immune response (Toll‐like receptor pathways, leukocyte proliferation, innate‐immune response, cellular response to TNF‐α, positive regulation of NF‐kappa B signalling). Visual combination of genes with related biological processes suggested functional interactions between biological processes related to activation of immune system (Figure 3D).

### 3.5 In vitro study: PCSK9 and endothelial activation/function

**FIGURE 4:**
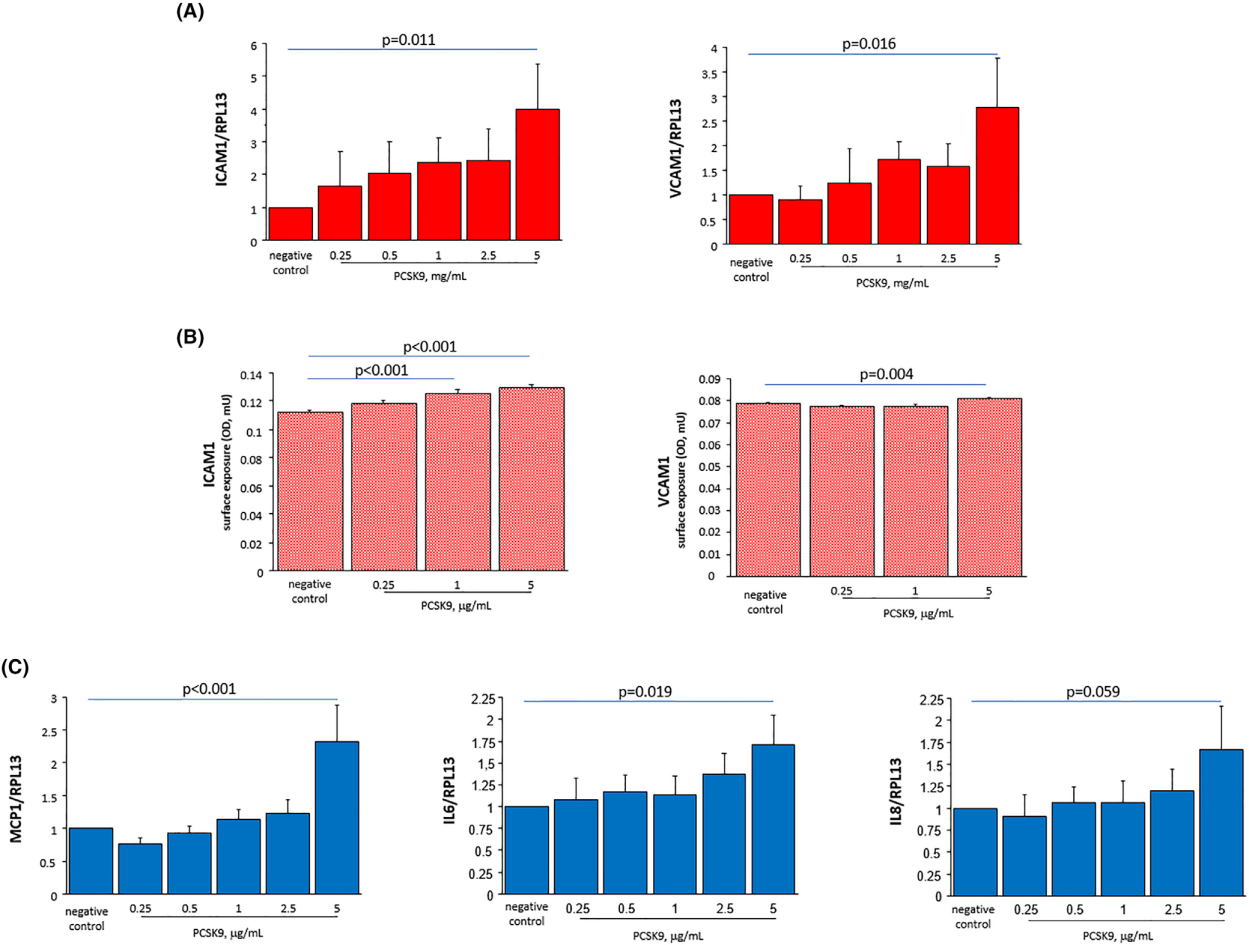
In vitro study. (A) HUVECs were treated with PCSK9 (.25–5 μg/mL) for 4 h and RT‐PCR was performed with specific primers for VCAM‐1, ICAM‐1, and RPL13a; (B) ICAM‐1 and VCAM‐1 surface exposure in HUVEC treated with PCSK9 (.25–5 μg/mL) overnight. At the end of the incubation time, VCAM‐1 and ICAM‐1 surface exposure was quantified by EIA. Values are mean ± SD of optical density arbitrary units (AU) at 405 nm; (C) HUVECs were treated with PCSK9 (.25–5 μg/mL) for 4 h and RT‐PCR was performed with specific primers for MCP‐1, IL6, IL8 and RPL13a.

After treatment with PCSK9, a significant increase in ICAM‐1 and VCAM‐1 was observed (Figure 4A) and the surface exposure of ICAM‐1 and VCAM‐1 was significantly up‐regulated (Figure 4B). Moreover, mRNA expression of MCP‐1 and IL6 was significantly upregulated after treatment with PCSK9 when compared with non‐treated cells, while no modification was observed for IL8 (Figure 4C).

## 4 DISCUSSION

The present study shows for the first time in patients with coronary atherosclerosis that plasma PCSK9 concentration was significantly associated with the progression of adverse coronary plaque features, investigated using serial coronary CTA. Patients with higher plasma PCSK9 had an increased progression of Fibrous‐fatty and Necrotic Core plaque volumes after a period of 6.4 ± 1.2 years. In particular, the association between plasma PCSK9 and the progression of Necrotic Core plaque volume was independent from baseline traditional cardiovascular risk factors and medications, including statins, supporting for PCSK9 an involvement in molecular pathways affecting residual atherosclerotic risk, beyond LDL‐C regulation. In addition, results from this study suggest that PCSK9 expression is related to the activity of inflammatory pathways that condition the adverse plaque phenotype progression (Graphical abstract).

Several clinical studies have investigated the relationship between circulating levels of PCSK9 and the presence and severity of ASCVD. Overall, these studies did not show clear or concordant results.^19^ On the other hand, in recent years, plaque stabilization and regression of atherosclerosis evaluated by CTA and intravascular ultrasound have been observed in patients treated with PCSK9 inhibitors, suggesting a relationship between PCSK9 and the progression of atherosclerosis.^20^, ^21^

Numerous preclinical studies have unequivocally demonstrated that the relationship between PCSK9 and atherosclerosis can be partially independent of hyperlipidaemia and may involve other effects relevant for atheroma formation and progression, such as those on inflammation and the endothelial system.^19^, ^22^, ^23^ In our study, the first indication of pathophysiological pathways involving PCSK9 in patients with CAD was obtained by the observation that PCSK9 plasma levels were associated not only with LDL‐C but also with IL‐6, MMP9 and ICAM1 plasma concentrations (Figure 1), suggesting a role for PCSK9 in processes related to inflammation and endothelial activation. As a matter of fact, the connection between circulating levels of PCSK9 and inflammatory markers such as hs‐CRP and IL6 had already been observed in previous studies,^24^, ^25^ even if in recent clinical trials in patients with CAD, PCSK9 inhibitors seemed not to affect systemic inflammation as determined by high sensitivity C‐reactive protein (CRP) levels.^26^

Our observations were indeed confirmed by the transcriptomic approach using whole blood from CAD patients at follow‐up. In the whole study group, plasma levels of PCSK9 were related to the expression of genes involved in inflammatory pathways as assessed by RNA‐seq (innate immunity, cytokine regulation, intracellular signal pathways such as Akt and MAPK).

Furthermore, in the 13% of study patients who express PCSK9 at the mRNA level in whole blood, a link between the mRNA expression of PCSK9 and of genes involved in the activation of the immune response (Toll‐like receptor pathways, leukocyte proliferation, innate‐immune response, cellular response to TNFa, cytokine production) was clearly documented. Importantly, the group of patients expressing PCSK9 also showed a significant increase in necrotic core PV and a significant decrease in fibrous PV at CTA at follow‐up. These observations suggest a pathophysiologic link between the expression of PCSK9, immune response, and an evolving adverse coronary atherosclerotic phenotype.^13^ Results from this study are in line with previous reports. In vitro studies reported that there is a stepwise increase in PCSK9 gene expression while transitioning from monocytes (PCSK9 being almost undetectable) to differentiating monocytes (PCSK9 significantly up‐regulated) to fully differentiated macrophages.^27^ Accumulating evidence suggests that PCSK9 may target receptors associated with inflammation other than the low‐density lipoprotein receptor (LDLR) and members of the LDLR family and could promote macrophage activation not only via lipid‐dependent mechanisms but also via lipid‐independent and LDLR‐dependent or ‐independent mechanisms.^22^ Specifically, it has been suggested that the role of PCSK9 in the positive regulation of inflammation and atherosclerotic lesion formation may involve the toll‐like receptor 4 (TLR4)/nuclear factor‐kappa B (NF‐kB) signalling pathway, which mediates the PCSK9‐induced expression of pro‐inflammatory cytokines and plays an important role in the initiation and development of atherosclerotic lesions by inducing vascular inflammation.^11^, ^19^, ^25^, ^28^, ^29^

Finally, we also explored the possible direct effects of PCSK9 on endothelial activation/inflammation by an in vitro approach. Treating EC with increased concentrations of PCSK9, an upregulation of ICAM‐1, VCAM‐1, and IL‐6 was observed, in line with previous studies in which PCSK9 administration induced LOX‐1 and VCAM‐1 expression in SMCs.^19^, ^22^, ^30^, ^31^

Limitations must be acknowledged in this study. Since whole blood samples were available only at follow‐up, we could not perform a transcriptomic analysis at baseline. The whole blood transcriptomic analysis does not inform cell type‐specific contribution. Information on statin therapy was limited and full data on types and dosages were not recorded. Due to the small sample size, further studies are necessary to confirm the role of PCSK9 in predicting adverse plaque progression in patients with CCS.

In conclusion, results from this study demonstrate, in a population of patients with stable CAD, that PCSK9 is independently associated with the progression of adverse coronary plaque phenotypes and with the activity of inflammatory pathways which may condition this progression, beyond LDL‐C regulation. Moreover, these results support the concept that pharmacologic regulation of PCSK9 may represent a cornerstone of cardiovascular prevention and offer some clues to understand some pleiotropic effects attributed to PCSK9 inhibitors. Specifically, the present results suggest that an approach based on the integration of PCSK9 inhibitors to first‐line lipid lowering therapies could be useful to specifically treat the inflammatory component of the residual atherosclerotic risk and prevent the progression of atherosclerosis.^32^ They raise the possibility that targeting PCSK9 may be beneficial even in individuals with low LDL cholesterol or who have already achieved LDL cholesterol lowering targets by other means.

Whether these effects are the result of actions of PCSK9 expressed locally in the cell types/tissues affected or due to circulating PCSK9 derived from the liver is not entirely clear, and further research will be needed to clarify this.

Finally, our study underlines the potentialities of integrating novel diagnostic tools, such as RNA‐seq of whole blood, for a personalised approach including the assessment of specific molecular inflammatory profiles to recognise individuals exhibiting a significant residual inflammatory ASCVD risk for targeted treatment.

## AUTHOR CONTRIBUTIONS

Rosetta Ragusa: Methodology, formal analysis, investigation, writing—original draft; Silvia Rocchiccioli: Investigation, data curation, writing—review and editing, funding acquisition; Serena Del Turco: Investigation, writing—review and editing; Antonio Morlando: Formal analysis, data curation; Giuseppina Basta: Writing—review and editing; Arthur Scholte: Methodology, investigation, data curation, writing—review and editing; Danilo Neglia: Conceptualization, writing—review and editing, funding acquisition; Chiara Caselli: Conceptualization, formal analysis, writing—original draft, supervision, funding acquisition.

## CONFLICT OF INTEREST STATEMENT

The authors declare that they have no conflict of interest.

## Supporting information


Appendix S1.

## Data Availability

The data underlying this article cannot be shared publicly due to the privacy of individuals that participated in the study. The data will be shared on reasonable request to the corresponding author.
