# Supplementary material for: PCSK9 and coronary atherosclerosis progression beyond LDL‐cholesterol in coronary artery disease patients
Source: Eur J Clin Invest. 2025 Jun 4;55(11):e70083. doi: 10.1111/eci.70083 (PMC12517242; doi:10.1111/eci.70083)
Supplement: Supplementary file 1 — Appendix S1. [file ECI-55-e70083-s001.docx]

**SUPPLEMENTAL MATERIALS**

**Soluble PCSK9 and coronary atherosclerosis progression beyond LDL-Cholesterol in coronary artery disease patients.**

Rosetta Ragusa,^1^ Silvia Rocchiccioli,^1^ Serena Del Turco,^1^ Antonio Morlando,^1^ Giuseppina Basta,^1^ Arthur Sholte,^3^ Danilo Neglia,^2^ Chiara Caselli^1,2^

^1^ CNR, Institute of Clinical Physiology, Pisa, Italy;

^2^ Fondazione Toscana G. Monasterio, Pisa, Italy.

^3^ Department of Cardiology, Heart Lung Center, Leiden University Medical Centre, Leiden, the Netherlands;

Address for correspondence: Chiara Caselli, PhD

CNR, Institute of Clinical Physiology

Via Moruzzi, 1 56100 Pisa, Italy

Fax: 390503152166

Phone: 390503153551

e-mail: [chiara.caselli@cnr.it](mailto:chiara.caselli@ifc.cnr.it)

**Figure S1.** Study flow-chart

**
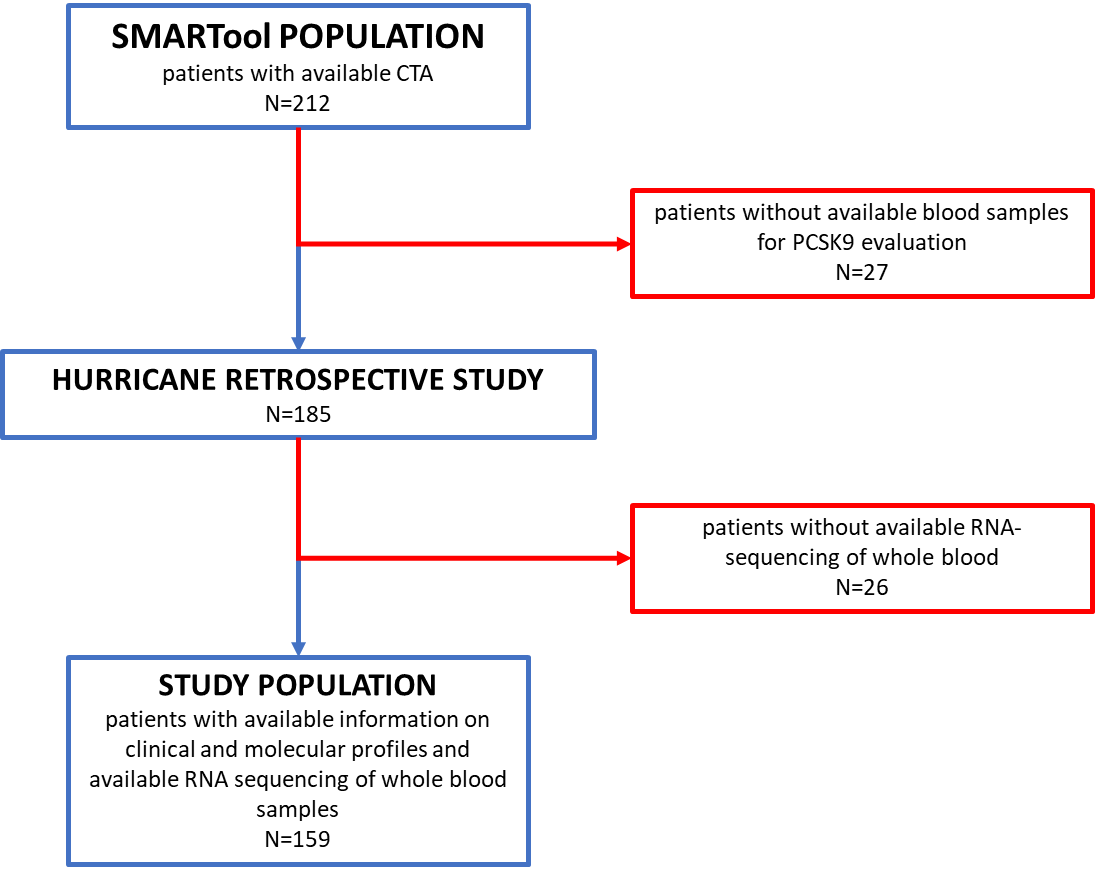
**

**Table S1. List of primers used in real Time PCR**

| **PRIMER PAIR** | **Gene Bank**  **accession number** | **sequence** |
| --- | --- | --- |
| **ICAM1** | NM_000201.3 | Forward: TGTCCCCCTCAAAAGTCATC  Reverse: TAGGCAACGGGGTCTCTATG |
| **VCAM1** | NM_001078.4 | Forward: GGACCACATCTACGCTGACA  Reverse: CACCTGGATTCCTTTTTCCA |
| **MCP1** | NM_002982.4 | Forward: CCCCAGTCACCTGCTGTTAT  Reverse: AGCTTCTTTGGGACACTTGC |
| **IL6** | NM_000600 | Forward: AGCGCCTTCGGTCCAGTTGC  Reverse: GTGGCTGTCTGTGTGGGGCG |
| **IL8** | NM_000584 | Forward: CCAAGCTGGCCGTGGCTTCTC  Reverse: TGTGTTGGCGCAGTGTGGTCC |
| **RPL13** | NM_012423 | Forward: CGCCCTACGACAAGAAAAAG  Reverse: CCGTAGCCTCATGAGCTGTT |

**Table S2. Baseline clinical and molecular features according to PCSK9 Tertiles.**

|  | **Tertile I**  **<153 ng/mL**  **n=53** | **Tertile II**  **153-234 ng/mL**  **n=53** | **Tertile III**  **>234 ng/mL**  **n=53** | ***p***  ***Value*** |
| --- | --- | --- | --- | --- |
| ***Clinical features*** |  |  |  |  |
| ***D*emographic** |  |  |  |  |
| Age, years | 61±8 | 64±7 | 64±7 | **0.031** |
| Male gender | 37 (70) | 31 (59) | 34 (64) | 0.478 |
| **Risk factors** |  |  |  |  |
| Family history | 22 (42) | 26 (49) | 22 (42) | 0.665 |
| Diabetes | 14 (26) | 12 (23) | 11 (21) | 0.782 |
| Hypertension | 34 (64) | 35 (66) | 37 (70) | 0.820 |
| Smoking | 10 (19) | 10 (19) | 6 (11) | 0.479 |
| Obesity | 17 (32) | 9 (17) | 6 (11) | **0.023** |
| **Medications** |  |  |  |  |
| Beta-blockers | 31 (58) | 22 (42) | 22 (42) | 0.130 |
| Calcium antagonists | 2 (4) | 9 (17) | 7 (13) | 0.087 |
| ACE inhibitors | 14 (26) | 18 (34) | 26 (49) | **0.048** |
| ARBs | 10 (19) | 4 (8) | 9 (17) | 0.207 |
| Diuretics | 10 (19) | 8 (15) | 12 (23) | 0.611 |
| Anti-diabetics | 12 (23) | 9 (17) | 5 (9) | 0.183 |
| Statins | 26 (49) | 33 (62) | 23 (43) | 0.137 |
| Anti-platelets | 41 (77) | 32 (60) | 35 (66) | 0.162 |
| ***Molecular Profile*** |  |  |  |  |
| Total-C, mg/dL | 169±41 | 180±49 | 203±51 | **0.003** |
| LDL-C, mg/dL | 100±32 | 106±43 | 123±46 | **0.052** |
| HDL-C, mg/dL | 46±13 | 50±14 | 57±16 | **0.001** |
| Triglycerides, mg/dL | 117±66 | 118±59 | 125±58 | 0.527 |
| FPG, mg/dL | 113±30 | 109±26 | 105±24 | 0.231 |
| insulin, μIU/ | 13±14 | 12±9 | 11±12 | 0.392 |
| hs-CRP, mg/dL | 034±0.47 | 0.29±0.40 | 0.48±0.73 | 0.274 |
| IL6, pg/mL | 0.82±0.78 | 1.03±1.18 | 1.44±1.57 | **0.024** |
| MMP9, ng/mL | 82±121 | 90±122 | 223±256 | **<0.001** |
| ICAM1, ng/mL | 187±70 | 200±69 | 208±82 | 0.311 |
| VCAM1, ng/mL | 507±92 | 540±189 | 564±150 | 0.129 |
| PCSK9, ng/mL | 114±28 | 195±24 | 350±133 | **<0.001** |

**Table S3.** **Univariate association of baseline PCSK9 and annual changes in plaque phenotypes at coronary CTA according to statins use.**

|  | ***No Statins*** | | | ***Statins*** | | |
| --- | --- | --- | --- | --- | --- | --- |
|  | ***Coefficient*** | ***SE*** | ***P value*** | ***Coefficient*** | ***SE*** | ***P value*** |
| **Annual change** |  |  |  |  |  |  |
| Total PV | 5.667 | 2.863 | ***0.051*** | 1.403 | 4.409 | 0.751 |
| Dense Calcium PV | -0.884 | 2.109 | 0.676 | -1.220 | 3.526 | 0.730 |
| Fibrous PV | 1.502 | 2.932 | 0.610 | -3.526 | 4.028 | 0.384 |
| Fibrous-Fatty PV | 3.034 | 1.216 | ***0.015*** | 1.105 | 1.245 | 0.378 |
| Necrotic Core PV | 3.853 | 1.814 | ***0.037*** | 5.793 | 2.528 | ***0.025*** |

**Bold** *P values* show statistically significant differences.

**Table S4. Univariate association of plasma PCSK9 at baseline and annual changes in plaque phenotypes at coronary CTA in females and males.**

|  | ***Woman*** | | | ***Man*** | | |
| --- | --- | --- | --- | --- | --- | --- |
|  | ***Coefficient*** | ***SE*** | ***P value*** | ***Coefficient*** | ***SE*** | ***P value*** |
| **Annual change** |  |  |  |  |  |  |
| Total PV | 6.315 | 2.470 | ***0.013*** | 3.513 | 3.811 | 0.165 |
| Dense Calcium PV | -0.553 | 2.173 | 0.800 | -0.174 | 2.811 | 0.951 |
| Fibrous PV | 3.826 | 2.037 | 0.066 | -4.751 | 3.770 | 0.210 |
| Fibrous-Fatty PV | 1.662 | 1.115 | 0.142 | 2.748 | 1.260 | ***0.032*** |
| Necrotic Core PV | 2.732 | 1.890 | 0.154 | 6.923 | 2.143 | ***0.002*** |

**Bold** *P values* show statistically significant differences.

**Table S5. List of genes significantly associated to plasma PCSK9 showing a P value ≤0.05 at RNA sequencing.**

| **Gene** | **Correlation** | **P value** |
| --- | --- | --- |
| *olah* | 0,31971092 | 4,4726E-05 |
| *daam2* | 0,31941094 | 4,5511E-05 |
| *flt3* | 0,31346395 | 6,4007E-05 |
| *pygm* | 0,27297603 | 0,00054223 |
| *adamts2* | 0,27145612 | 0,00058395 |
| *areg* | 0,27075324 | 0,00060423 |
| *c7orf25* | 0,27016294 | 0,00062176 |
| *gcm1* | 0,26855628 | 0,00067188 |
| *prrx2* | 0,26826272 | 0,00068143 |
| *hal* | 0,26715575 | 0,00071857 |
| *adam11* | 0,26475695 | 0,00080553 |
| *btbd19* | 0,26328946 | 0,0008634 |
| *sec61a2* | 0,2613906 | 0,00094394 |
| *clul1* | 0,26096598 | 0,00096286 |
| *rp11_47i22_3* | 0,26044658 | 0,00098649 |
| *ddtl* | 0,25956581 | 0,00102777 |
| *fkbp5* | 0,25915435 | 0,00104759 |
| *acpl2* | 0,25779997 | 0,00111534 |
| *mgp* | 0,25323614 | 0,00137423 |
| *prcd* | 0,25299903 | 0,00138908 |
| *epcam* | 0,25087668 | 0,00152861 |
| *higd1c* | 0,2482786 | 0,00171676 |
| *rgpd2* | 0,24797679 | 0,00173993 |
| *ddit4* | 0,24787368 | 0,00174792 |
| *klrf2* | 0,24752764 | 0,00177495 |
| *armc12* | 0,24677885 | 0,00183475 |
| *sfrp1* | 0,24384035 | 0,00208755 |
| *prkdc* | 0,24375268 | 0,00209556 |
| *cryba1* | 0,24339184 | 0,0021288 |
| *npipa2* | 0,24339184 | 0,0021288 |
| *tpst1* | 0,24160096 | 0,00230102 |
| *efna1* | 0,24110972 | 0,00235041 |
| *uroc1* | 0,23925953 | 0,00254521 |
| *arhgef40* | 0,23771997 | 0,00271832 |
| *prame* | 0,23600181 | 0,00292403 |
| *smap2* | 0,23545196 | 0,00299278 |
| *rp1l1* | 0,23460397 | 0,00310167 |
| *kiaa0319* | 0,23403721 | 0,00317642 |
| *helt* | 0,23379696 | 0,0032086 |
| *amn* | 0,2307434 | 0,00364398 |
| *tsc22d3* | 0,22731344 | 0,00419589 |
| *ankrd55* | 0,22608634 | 0,00441088 |
| *gtf2ird2* | 0,22547639 | 0,00452138 |
| *ube3d* | 0,22488431 | 0,00463102 |
| *il18r1* | 0,22459767 | 0,00468494 |
| *ucn2* | 0,22326053 | 0,00494402 |
| *st6galnac3* | 0,22274967 | 0,00504634 |
| *scnn1a* | 0,22244976 | 0,00510729 |
| *slc51b* | 0,22233839 | 0,00513009 |
| *golga6l19* | 0,22088906 | 0,00543523 |
| *tp53tg3c* | 0,22088906 | 0,00543523 |
| *lrrc37a3* | 0,22086713 | 0,00543997 |
| *tlr2* | 0,21991981 | 0,00564824 |
| *ccdc183* | 0,21970497 | 0,00569645 |
| *rp11_231c14_4* | 0,21918881 | 0,00581379 |
| *ac140481_2* | 0,21911152 | 0,00583154 |
| *fam174b* | 0,21866808 | 0,00593435 |
| *rbm11* | 0,21787257 | 0,00612284 |
| *kcne1* | 0,21712755 | 0,00630419 |
| *c12orf55* | 0,21660854 | 0,00643333 |
| *bnipl* | 0,2157923 | 0,00664121 |
| *vsig4* | 0,21572186 | 0,00665943 |
| *c17orf53* | 0,21528606 | 0,00677313 |
| *c16orf96* | 0,21523079 | 0,00678767 |
| *rtdr1* | 0,21356638 | 0,00723883 |
| *rasgrp2* | 0,21353343 | 0,00724802 |
| *htra1* | 0,21329117 | 0,00731594 |
| *nsmaf* | 0,21309018 | 0,00737272 |
| *dmrtc2* | 0,21278651 | 0,00745924 |
| *krtap10_9* | 0,21202907 | 0,007679 |
| *msl3* | 0,21171507 | 0,00777177 |
| *pfkfb2* | 0,21145683 | 0,00784882 |
| *spata6* | 0,20974075 | 0,00837823 |
| *slc5a9* | 0,20968238 | 0,00839678 |
| *leap2* | 0,20899372 | 0,0086184 |
| *grb10* | 0,20858347 | 0,00875288 |
| *cabp1* | 0,20834468 | 0,008832 |
| *ac083862_1* | 0,20831031 | 0,00884344 |
| *mapk13* | 0,20781677 | 0,00900918 |
| *hcg27* | 0,20731506 | 0,00918046 |
| *c12orf79* | 0,20703052 | 0,00927888 |
| *lsmem1* | 0,20607303 | 0,00961694 |
| *dct* | 0,20569113 | 0,00975478 |
| *col9a2* | 0,20558924 | 0,00979185 |
| *nsun7* | 0,205108 | 0,00996862 |
| *ac124890_1* | 0,20459525 | 0,01016005 |
| *gtf2ird2b* | 0,20297233 | 0,0107875 |
| *sts* | 0,20286717 | 0,01082931 |
| *tenm1* | 0,20238618 | 0,01102236 |
| *exoc6* | 0,20168643 | 0,01130862 |
| *znf630* | 0,20148671 | 0,01139151 |
| *efcab1* | 0,20145748 | 0,01140369 |
| *fam27e1* | 0,20142188 | 0,01141854 |
| *nbpf14* | 0,20103236 | 0,01158208 |
| *c4a* | 0,20065151 | 0,01174398 |
| *gal3st4* | 0,19981958 | 0,01210455 |
| *btg1* | 0,1996213 | 0,0121919 |
| *apba2* | 0,19927405 | 0,01234623 |
| *exo5* | 0,19854519 | 0,01267573 |
| *zrsr1* | 0,19830487 | 0,01278605 |
| *knstrn* | 0,19790893 | 0,01296964 |
| *ccdc17* | 0,19790239 | 0,0129727 |
| *znf589* | 0,19722125 | 0,01329401 |
| *rnase13* | 0,19681309 | 0,01348987 |
| *43161_2* | 0,19678407 | 0,01350389 |
| *per1* | 0,19665698 | 0,01356544 |
| *lcn12* | 0,19635741 | 0,01371151 |
| *cyth2* | 0,19550584 | 0,01413426 |
| *ces3* | 0,19534166 | 0,01421706 |
| *ly6g5b* | 0,1952145 | 0,01428149 |
| *c1orf174* | 0,1951734 | 0,01430236 |
| *pias2* | 0,19509823 | 0,01434061 |
| *c20orf62* | 0,19494302 | 0,01441988 |
| *rp11_295p9_3* | 0,19408856 | 0,01486313 |
| *hecw2* | 0,19352005 | 0,01516459 |
| *c9orf156* | 0,1934519 | 0,01520108 |
| *kcnq2* | 0,19329327 | 0,01528632 |
| *klhl2* | 0,19292906 | 0,01548361 |
| *linc01118* | 0,1922242 | 0,01587173 |
| *cntnap3* | 0,19197966 | 0,01600835 |
| *golga6l4* | 0,19161848 | 0,016212 |
| *neb* | 0,19159528 | 0,01622516 |
| *fam19a2* | 0,19136397 | 0,01635686 |
| *irs2* | 0,19124686 | 0,01642389 |
| *pipox* | 0,19098361 | 0,01657544 |
| *cds2* | 0,19087651 | 0,01663745 |
| *fchsd1* | 0,19077509 | 0,01669635 |
| *foxq1* | 0,19074705 | 0,01671267 |
| *gpr176* | 0,19071427 | 0,01673176 |
| *wtap* | 0,1905252 | 0,01684226 |
| *rab36* | 0,18989896 | 0,01721279 |
| *il13ra1* | 0,18951847 | 0,01744135 |
| *ccdc114* | 0,18932504 | 0,01755855 |
| *kazn* | 0,18887451 | 0,01783419 |
| *rp11_683l23_1* | 0,18862171 | 0,0179905 |
| *cpsf7* | 0,18860472 | 0,01800104 |
| *fndc7* | 0,18847377 | 0,01808251 |
| *ergic1* | 0,18833248 | 0,01817078 |
| *dmrt2* | 0,18801777 | 0,01836872 |
| *mapre3* | 0,1879563 | 0,0184076 |
| *al135998_1* | 0,18778391 | 0,01851702 |
| *rundc3b* | 0,18758109 | 0,01864648 |
| *dlx4* | 0,18729758 | 0,01882875 |
| *fhit* | 0,18724092 | 0,01886536 |
| *gpr157* | 0,18707188 | 0,01897495 |
| *c3orf62* | 0,18685091 | 0,01911905 |
| *ankzf1* | 0,1862656 | 0,01950531 |
| *c22orf15* | 0,18598172 | 0,01969507 |
| *smoc2* | 0,18571223 | 0,01987668 |
| *aktip* | 0,18543603 | 0,02006432 |
| *mdm2* | 0,18527638 | 0,02017348 |
| *hsd17b7* | 0,18501258 | 0,02035497 |
| *dnhd1* | 0,18486897 | 0,02045436 |
| *amph* | 0,18433985 | 0,02082419 |
| *pfkm* | 0,18432379 | 0,02083551 |
| *inhbb* | 0,18410581 | 0,02098961 |
| *ctc_487m23_8* | 0,18380552 | 0,0212035 |
| *tmem120b* | 0,18367899 | 0,0212942 |
| *c_xorf58* | 0,1836466 | 0,02131746 |
| *pik3ip1* | 0,18359544 | 0,02135427 |
| *kiaa1257* | 0,18341798 | 0,02148234 |
| *rp11_89n17_1* | 0,18340458 | 0,02149203 |
| *cachd1* | 0,18301358 | 0,02177665 |
| *znf33a* | 0,1829725 | 0,02180675 |
| *wls* | 0,18282698 | 0,02191363 |
| *camk1d* | 0,18218834 | 0,02238806 |
| *kb_1980e6_3* | 0,18216795 | 0,02240335 |
| *itgax* | 0,18213457 | 0,02242841 |
| *vipr1* | 0,18193449 | 0,02257908 |
| *tuft1* | 0,18137019 | 0,02300874 |
| *echdc3* | 0,18115566 | 0,02317392 |
| *col11a2* | 0,18113385 | 0,02319077 |
| *cyb561d1* | 0,18080583 | 0,02344547 |
| *ufd1l* | 0,18078757 | 0,02345972 |
| *ccnd3* | 0,18078459 | 0,02346205 |
| *inpp5a* | 0,18060441 | 0,02360305 |
| *st6galnac2* | 0,18037653 | 0,02378243 |
| *myh3* | 0,18027431 | 0,02386327 |
| *crhr1* | 0,18011764 | 0,02398764 |
| *c10orf105* | 0,17975401 | 0,02427844 |
| *manba* | 0,17927133 | 0,02466911 |
| *kcnh3* | 0,17768278 | 0,02599318 |
| *ssbp3_as1* | 0,17739878 | 0,02623619 |
| *dync1i1* | 0,17737708 | 0,02625484 |
| *ing5* | 0,17695417 | 0,02662054 |
| *dcst2* | 0,17675188 | 0,026797 |
| *adam17* | 0,17672094 | 0,02682409 |
| *sln* | 0,17666068 | 0,02687689 |
| *pou6f1* | 0,17643868 | 0,02707219 |
| *rab43* | 0,17625553 | 0,02723423 |
| *lrtm1* | 0,17623244 | 0,02725471 |
| *alox15b* | 0,17590206 | 0,02754929 |
| *lsmem2* | 0,17557021 | 0,02784792 |
| *tmem45b* | 0,17537113 | 0,02802838 |
| *prom2* | 0,17525798 | 0,0281314 |
| *fam45a* | 0,17522903 | 0,02815781 |
| *ttll10* | 0,17501279 | 0,02835574 |
| *tdp2* | 0,17469491 | 0,02864886 |
| *rbm5* | 0,17436075 | 0,02895977 |
| *kcnj14* | 0,17434346 | 0,02897594 |
| *dfnb59* | 0,17432555 | 0,02899269 |
| *c10orf91* | 0,17396192 | 0,02933462 |
| *pigb* | 0,17373188 | 0,0295527 |
| *asph* | 0,17329825 | 0,02996754 |
| *hyal1* | 0,17287045 | 0,03038165 |
| *snrnp70* | 0,1727214 | 0,03052708 |
| *tceal2* | 0,17268928 | 0,03055849 |
| *rgpd1* | 0,17254305 | 0,03070186 |
| *htr6* | 0,17244088 | 0,03080237 |
| *ccbl1* | 0,17229028 | 0,03095104 |
| *trim39* | 0,17220257 | 0,0310379 |
| *dsn1* | 0,17194179 | 0,03129738 |
| *slc31a2* | 0,1718514 | 0,03138775 |
| *cdk5r1* | 0,17124659 | 0,03199815 |
| *fam166b* | 0,17084362 | 0,03241041 |
| *rnf144b* | 0,17077156 | 0,03248461 |
| *tmem143* | 0,17075014 | 0,0325067 |
| *znf487* | 0,17056753 | 0,03269547 |
| *brd8* | 0,17041391 | 0,03285499 |
| *u2af1l4* | 0,17035305 | 0,03291838 |
| *ralgapa2* | 0,17025947 | 0,03301604 |
| *cdc42se1* | 0,17017429 | 0,03310514 |
| *slc9a8* | 0,16983163 | 0,03346566 |
| *amy2b* | 0,16980545 | 0,03349334 |
| *pten* | 0,16962559 | 0,03368404 |
| *duoxa2* | 0,16952712 | 0,03378883 |
| *cytip* | 0,16946955 | 0,03385022 |
| *rasgrf2* | 0,16909461 | 0,03425237 |
| *col7a1* | 0,16899042 | 0,03436484 |
| *btnl9* | 0,16891414 | 0,03444738 |
| *tead3* | 0,16842761 | 0,03497779 |
| *igf1r* | 0,16835663 | 0,03505574 |
| *fam169b* | 0,16821237 | 0,03521462 |
| *slc35e3* | 0,16820471 | 0,03522308 |
| *tmed8* | 0,16800185 | 0,03544759 |
| *far2* | 0,16760249 | 0,0358931 |
| *slc16a5* | 0,16728702 | 0,03624835 |
| *nfkbia* | 0,16723374 | 0,03630864 |
| *gstm2* | 0,16716373 | 0,036388 |
| *slc29a2* | 0,16708189 | 0,03648095 |
| *hspg2* | 0,16701781 | 0,03655386 |
| *dnmt3a* | 0,16693756 | 0,03664535 |
| *rfx2* | 0,16688552 | 0,03670478 |
| *fuz* | 0,16683441 | 0,03676323 |
| *ezh1* | 0,16676245 | 0,03684566 |
| *dusp15* | 0,16669308 | 0,03692526 |
| *prpf40b* | 0,16662363 | 0,0370051 |
| *ceacam19* | 0,16660083 | 0,03703135 |
| *fam65b* | 0,16645983 | 0,03719399 |
| *prmt2* | 0,16636821 | 0,0373 |
| *atg16l2* | 0,16624361 | 0,03744457 |
| *ttc9* | 0,16622817 | 0,03746252 |
| *flj00273* | 0,16593075 | 0,03780967 |
| *slc38a2* | 0,16577526 | 0,03799223 |
| *sncb* | 0,16568216 | 0,0381019 |
| *them4* | 0,16567512 | 0,0381102 |
| *c6orf229* | 0,16549971 | 0,03831759 |
| *ppap2b* | 0,16529245 | 0,03856385 |
| *cnrip1* | 0,16491894 | 0,03901101 |
| *pkp4* | 0,16488741 | 0,03904896 |
| *tex12* | 0,1645445 | 0,03946366 |
| *tle2* | 0,16421636 | 0,03986396 |
| *vapb* | 0,16407607 | 0,04003614 |
| *klhdc9* | 0,16399963 | 0,04013022 |
| *ctb_50l17_14* | 0,16395748 | 0,04018217 |
| *nqo2* | 0,16384545 | 0,04032054 |
| *fam212b* | 0,16384116 | 0,04032584 |
| *ccdc19* | 0,16353067 | 0,04071145 |
| *vsig8* | 0,16347851 | 0,04077654 |
| *ssh2* | 0,16341752 | 0,04085275 |
| *tial1* | 0,1633132 | 0,04098338 |
| *cyyr1* | 0,16325294 | 0,04105899 |
| *rnaset2* | 0,16324337 | 0,04107102 |
| *trim27* | 0,16298611 | 0,04139525 |
| *frat1* | 0,16295583 | 0,04143355 |
| *rem2* | 0,16295445 | 0,04143529 |
| *dgkd* | 0,16288312 | 0,04152564 |
| *prdm15* | 0,1627845 | 0,04165083 |
| *arhgap19* | 0,16276626 | 0,04167402 |
| *fam46b* | 0,1625175 | 0,04199135 |
| *hsdl1* | 0,16239029 | 0,0421544 |
| *ambp* | 0,16225411 | 0,04232955 |
| *cyth1* | 0,16220757 | 0,04238955 |
| *cflar* | 0,16215426 | 0,04245836 |
| *mcl1* | 0,16203364 | 0,04261438 |
| *dcaf4l1* | 0,16190489 | 0,04278147 |
| *hsbp1l1* | 0,16189658 | 0,04279227 |
| *plxdc1* | 0,1618719 | 0,04282436 |
| *mtrnr2l8* | 0,16136649 | 0,04348612 |
| *fam86b2* | 0,16103095 | 0,04393015 |
| *rfpl4al1* | 0,16071327 | 0,04435405 |
| *plin5* | 0,16032426 | 0,04487777 |
| *acer1* | 0,16023344 | 0,04500077 |
| *c3orf80* | 0,16011357 | 0,04516357 |
| *asprv1* | 0,16007554 | 0,04521532 |
| *clk1* | 0,16005823 | 0,04523889 |
| *krtdap* | 0,15987324 | 0,04549143 |
| *rp1_102h19_8* | 0,15979819 | 0,04559421 |
| *ppp4r1* | 0,15975973 | 0,04564696 |
| *nudt5* | 0,15975935 | 0,04564749 |
| *adamtsl4_as1* | 0,15966846 | 0,04577236 |
| *hs3st3b1* | 0,15957235 | 0,0459047 |
| *aph1b* | 0,15956941 | 0,04590875 |
| *mak* | 0,15954586 | 0,04594124 |
| *znf608* | 0,15948959 | 0,04601891 |
| *csgalnact1* | 0,15938038 | 0,04617 |
| *asb8* | 0,1593201 | 0,04625357 |
| *armc2* | 0,1592869 | 0,04629965 |
| *irak3* | 0,15914763 | 0,04649338 |
| *zc3h12b* | 0,15870628 | 0,04711176 |
| *pbxip1* | 0,15809445 | 0,04798032 |
| *herc2* | 0,15767486 | 0,04858366 |
| *gper1* | 0,15736722 | 0,04903001 |
| *birc7* | 0,15723485 | 0,04922311 |
| *ephb6* | 0,15719921 | 0,04927521 |
| *t* | 0,15713057 | 0,04937568 |
| *tmem253* | 0,15712594 | 0,04938245 |
| *espnl* | 0,15682726 | 0,04982167 |
| *zc2hc1b* | 0,156743 | 0,04994616 |
| *anxa5* | -0,1567136 | 0,0499897 |
| *gspt2* | -0,156796 | 0,04986777 |
| *rhov* | -0,1569791 | 0,04959797 |
| *tgfbi* | -0,1575049 | 0,04882979 |
| *ap003774_4* | -0,1575335 | 0,04878838 |
| *dnajc15* | -0,1575358 | 0,04878501 |
| *adamdec1* | -0,1576459 | 0,04862559 |
| *acsl5* | -0,1576462 | 0,04862508 |
| *mlxipl* | -0,1579125 | 0,04824122 |
| *tmem51* | -0,1580377 | 0,04806155 |
| *tceb3* | -0,1580948 | 0,04797976 |
| *hivep3* | -0,1585928 | 0,04727185 |
| *exo1* | -0,1587087 | 0,0471083 |
| *cpvl* | -0,1587984 | 0,04698218 |
| *dusp10* | -0,1589461 | 0,0467749 |
| *gnpda1* | -0,1590957 | 0,04656573 |
| *odc1* | -0,1592158 | 0,04639842 |
| *spsb1* | -0,159306 | 0,0462731 |
| *rhno1* | -0,1593464 | 0,04621703 |
| *hla_dra* | -0,1593853 | 0,04616319 |
| *adap2* | -0,1594498 | 0,04607395 |
| *zfp57* | -0,1595678 | 0,04591102 |
| *c21orf62* | -0,1597693 | 0,04563389 |
| *cd300lb* | -0,159803 | 0,04558757 |
| *fam109b* | -0,1598515 | 0,04552119 |
| *tceanc* | -0,1598574 | 0,04551315 |
| *pnp* | -0,1601258 | 0,04514693 |
| *tlr3* | -0,1603457 | 0,04484877 |
| *plk5* | -0,1603669 | 0,04482007 |
| *tmem106a* | -0,1604053 | 0,04476822 |
| *tmem52b* | -0,1604225 | 0,04474507 |
| *zbed2* | -0,160435 | 0,04472813 |
| *rrm2* | -0,1604598 | 0,04469465 |
| *ptprf* | -0,160506 | 0,0446325 |
| *tm7sf3* | -0,1608469 | 0,04417531 |
| *eif2b1* | -0,1608848 | 0,0441248 |
| *ctsb* | -0,161449 | 0,04337746 |
| *mlec* | -0,1614818 | 0,04333444 |
| *caps2* | -0,1616807 | 0,0430737 |
| *erp44* | -0,1619578 | 0,04271278 |
| *znf221* | -0,1620763 | 0,04255909 |
| *fbp1* | -0,1625821 | 0,04190874 |
| *slc27a2* | -0,1625912 | 0,04189707 |
| *pnpo* | -0,1628616 | 0,04155294 |
| *hla_dmb* | -0,1631745 | 0,04115764 |
| *tubal3* | -0,1633355 | 0,04095545 |
| *fam47e_stbd1_2* | -0,1634003 | 0,04087423 |
| *znf676* | -0,1635105 | 0,04073664 |
| *csf1r* | -0,1638353 | 0,04033314 |
| *camkv* | -0,1640122 | 0,04011476 |
| *hyou1* | -0,1641152 | 0,03998805 |
| *zfp41_2* | -0,1642118 | 0,03986952 |
| *c17orf72* | -0,1644432 | 0,03958686 |
| *ttyh2* | -0,1645039 | 0,03951299 |
| *haao* | -0,1646538 | 0,0393311 |
| *qrfp* | -0,1648049 | 0,03914842 |
| *inhbe* | -0,1648628 | 0,03907859 |
| *cmbl* | -0,1648796 | 0,03905835 |
| *tnfsf13* | -0,164947 | 0,03897723 |
| *nkx3_2* | -0,1651626 | 0,03871877 |
| *tspan4* | -0,1652014 | 0,03867242 |
| *tmprss15* | -0,1652093 | 0,03866307 |
| *mybl2* | -0,1656993 | 0,03808175 |
| *rbl1_2* | -0,1658699 | 0,03788098 |
| *mica* | -0,1660596 | 0,03765896 |
| *lgals9c* | -0,1660682 | 0,03764885 |
| *slc6a12* | -0,166199 | 0,03749646 |
| *cecr1* | -0,1662153 | 0,03747753 |
| *slc6a3* | -0,1663 | 0,03737903 |
| *abcg2* | -0,1666581 | 0,0369654 |
| *slc46a1* | -0,1666638 | 0,03695885 |
| *a2m* | -0,1668859 | 0,03670436 |
| *pdia6* | -0,1669059 | 0,03668145 |
| *mael* | -0,1671106 | 0,03644835 |
| *dgkq* | -0,1672529 | 0,03628696 |
| *esyt3* | -0,1673782 | 0,03614536 |
| *rap2b* | -0,1674517 | 0,03606255 |
| *lrp1* | -0,1674718 | 0,03603987 |
| *capn11* | -0,1674792 | 0,03603164 |
| *epb41l3* | -0,1674907 | 0,03601864 |
| *spa17* | -0,1676231 | 0,03586993 |
| *slc35d2* | -0,1676596 | 0,03582911 |
| *slc9a9* | -0,167841 | 0,03562645 |
| *bub1b* | -0,1678852 | 0,03557718 |
| *dtx4* | -0,1679848 | 0,03546655 |
| *tmsb4y* | -0,1680368 | 0,03540883 |
| *nrep* | -0,1683857 | 0,03502378 |
| *sulf2* | -0,1684615 | 0,03494062 |
| *tubb3_2* | -0,1693715 | 0,03395497 |
| *ostn* | -0,169467 | 0,03385298 |
| *dennd1a* | -0,1704039 | 0,03286538 |
| *rab38* | -0,1705282 | 0,03273624 |
| *mmaa* | -0,1707273 | 0,03253025 |
| *tigit* | -0,171086 | 0,03216194 |
| *tor3a* | -0,1710934 | 0,03215439 |
| *cd68* | -0,1713627 | 0,03188018 |
| *znf385a* | -0,1714093 | 0,03183294 |
| *bub1* | -0,1717228 | 0,03151674 |
| *lrfn4* | -0,1718568 | 0,03138235 |
| *ak4* | -0,1719443 | 0,03129492 |
| *ccr10* | -0,1722 | 0,03104046 |
| *x_xbac_bpg181m17_5* | -0,1729285 | 0,03032516 |
| *cd300e* | -0,1734841 | 0,02978911 |
| *mag* | -0,1738241 | 0,02946512 |
| *rp11_1035h13_3* | -0,1742519 | 0,02906168 |
| *pfdn4* | -0,1745141 | 0,02881669 |
| *osr2* | -0,1747502 | 0,02859771 |
| *il15ra* | -0,1748556 | 0,02850038 |
| *ccdc146* | -0,1754334 | 0,02797182 |
| *atf3* | -0,1756022 | 0,02781902 |
| *rp11_944c7_1* | -0,1757838 | 0,02765544 |
| *anks1b* | -0,1758627 | 0,02758453 |
| *cpsf1* | -0,1758656 | 0,027582 |
| *c12orf5* | -0,1758744 | 0,02757409 |
| *parpbp* | -0,1759077 | 0,02754428 |
| *efnb1* | -0,1759315 | 0,02752289 |
| *fzd5* | -0,1761716 | 0,02730877 |
| *rgs1* | -0,1762414 | 0,02724675 |
| *arhgap39* | -0,1765787 | 0,02694888 |
| *txndc15* | -0,1765834 | 0,02694472 |
| *ttyh3* | -0,1774806 | 0,02616598 |
| *oaz3* | -0,1775177 | 0,02613419 |
| *hnmt* | -0,177554 | 0,02610309 |
| *cdca2* | -0,1775885 | 0,02607365 |
| *tlr7* | -0,1776582 | 0,02601413 |
| *plxnb2* | -0,177743 | 0,02594191 |
| *clec4g* | -0,1777491 | 0,02593667 |
| *ctc_241n9_1* | -0,1780572 | 0,02567577 |
| *fam47e_stbd1* | -0,1780783 | 0,02565796 |
| *kcnc3* | -0,1782009 | 0,02555476 |
| *chrnb1* | -0,1785601 | 0,02525461 |
| *vps33a* | -0,1787121 | 0,02512851 |
| *ncaph* | -0,1788064 | 0,02505054 |
| *spem1* | -0,1794799 | 0,02449965 |
| *wdr63* | -0,1796251 | 0,02438228 |
| *smtnl1* | -0,1798878 | 0,0241711 |
| *limk1* | -0,1802651 | 0,02387058 |
| *pid1* | -0,1806228 | 0,02358866 |
| *rd3l* | -0,1806688 | 0,02355254 |
| *tmem241* | -0,1808516 | 0,0234098 |
| *nusap1* | -0,1809956 | 0,02329782 |
| *ppp1r17* | -0,1812619 | 0,02309196 |
| *sftpb* | -0,1816034 | 0,0228303 |
| *slc39a11* | -0,1816549 | 0,02279108 |
| *eif1ay* | -0,1819113 | 0,02259663 |
| *tox2* | -0,1819875 | 0,02253911 |
| *mki67* | -0,1825905 | 0,02208826 |
| *tyms* | -0,1828432 | 0,02190173 |
| *sdc3* | -0,1835988 | 0,02135187 |
| *inhba* | -0,1836919 | 0,02128495 |
| *or5b12* | -0,1839896 | 0,02107219 |
| *pax8* | -0,1842154 | 0,020912 |
| *bysl* | -0,1843598 | 0,02081016 |
| *ca2* | -0,1843803 | 0,02079575 |
| *slc37a2* | -0,184799 | 0,02050295 |
| *fanca* | -0,184815 | 0,0204918 |
| *kiaa0101* | -0,185564 | 0,01997722 |
| *tex9* | -0,1861754 | 0,01956546 |
| *slc25a35* | -0,1863045 | 0,01947946 |
| *rgag1* | -0,1865593 | 0,01931064 |
| *naga* | -0,1866328 | 0,01926221 |
| *myoz3* | -0,1887396 | 0,01791749 |
| *rp11_166b2_1* | -0,1892381 | 0,01761143 |
| *rad51* | -0,1893252 | 0,01755845 |
| *foxp3* | -0,1897796 | 0,01728418 |
| *dio1* | -0,189834 | 0,01725165 |
| *bcl2l13* | -0,1902067 | 0,01702982 |
| *c1orf198* | -0,1903926 | 0,01692015 |
| *dhdds* | -0,1919721 | 0,01601257 |
| *clspn* | -0,1920641 | 0,01596104 |
| *specc1* | -0,1924631 | 0,01573925 |
| *c3orf52* | -0,1926524 | 0,01563492 |
| *ceacam1* | -0,1927601 | 0,01557586 |
| *rp11_343c2_9* | -0,1933244 | 0,01526959 |
| *fam72b* | -0,1938462 | 0,01499102 |
| *gpr161* | -0,1939445 | 0,01493904 |
| *cpt2* | -0,1944554 | 0,0146714 |
| *cenpe* | -0,1948324 | 0,01447661 |
| *agpat3* | -0,1950273 | 0,01437678 |
| *slc1a5* | -0,1959648 | 0,013905 |
| *cdkn2c* | -0,1961688 | 0,01380417 |
| *tpmt* | -0,1963206 | 0,01372957 |
| *prkg2* | -0,1963547 | 0,01371286 |
| *cox17* | -0,1969408 | 0,01342829 |
| *stard8* | -0,1970548 | 0,01337357 |
| *atp1b1* | -0,1978977 | 0,01297491 |
| *tlcd1* | -0,1985204 | 0,01268705 |
| *fank1* | -0,1993125 | 0,01232906 |
| *fxn* | -0,2000282 | 0,01201325 |
| *mafb* | -0,2000614 | 0,01199874 |
| *fxyd4* | -0,2006744 | 0,01173419 |
| *pea15* | -0,2010333 | 0,01158166 |
| *tpp1* | -0,2015834 | 0,0113513 |
| *naaa* | -0,2017173 | 0,01129586 |
| *col10a1* | -0,2023824 | 0,0110239 |
| *znf174* | -0,2039837 | 0,01039262 |
| *sbspon* | -0,2046285 | 0,01014753 |
| *hdx* | -0,2056307 | 0,00977677 |
| *gcnt2* | -0,2062149 | 0,00956616 |
| *nup62* | -0,2067059 | 0,00939229 |
| *atf5* | -0,2069379 | 0,00931111 |
| *mpeg1* | -0,2078238 | 0,0090068 |
| *casz1* | -0,2091651 | 0,00856276 |
| *rnf214* | -0,209755 | 0,00837372 |
| *arhgef10l* | -0,2100089 | 0,00829348 |
| *slc37a1* | -0,2121695 | 0,00763782 |
| *rbfox3* | -0,2138816 | 0,00715139 |
| *trim26* | -0,2145922 | 0,00695773 |
| *depdc1* | -0,2156616 | 0,00667506 |
| *chaf1a* | -0,2178745 | 0,00612238 |
| *c15orf56* | -0,2190018 | 0,00585682 |
| *nrros* | -0,2215257 | 0,00529926 |
| *ccrl2* | -0,2218622 | 0,0052286 |
| *ctsl* | -0,2234246 | 0,00491156 |
| *ccl3l1* | -0,2239024 | 0,0048181 |
| *src* | -0,2287612 | 0,00395436 |
| *fuca1* | -0,2300868 | 0,00374427 |
| *shisa3* | -0,2370604 | 0,00279569 |
| *gtf2h2* | -0,2415353 | 0,00230756 |

**Table S6. Clinical, molecular and coronary CTA data of the study population according to mRNA expression of *PCSK9* at Follow up.**

|  | ***NO***  ***PCSK9 expression***  ***n=139*** | ***YES***  ***PCSK9 expression***  ***n=20*** | ***p***  ***Value*** |
| --- | --- | --- | --- |
| ***Clinical features*** |  |  |  |
| ***D*emographic** |  |  |  |
| Age, years | 69±8 | 72±7 | **<0.001** |
| Male gender | 90 (65) | 12 (60) | 0.679 |
| **Risk factors** |  |  |  |
| Family history | 66 (47) | 4 (20) | **0.021** |
| Diabetes | 42 (30) | 4 (20) | 0.346 |
| Hypertension | 106 (76) | 16 (80) | 0.711 |
| Smoking | 14 (10) | 3 (15) | 0.505 |
| Obesity | 36 (26) | 5 (25) | 0.091 |
| **Medications** |  |  |  |
| Beta-blockers | 72 (52) | 11 (55) | 0.789 |
| Calcium antagonists | 33 (24) | 2 (10) | 0.166 |
| ACE inhibitors | 58 (42) | 8 (40) | 0.884 |
| ARBs | 25 (18) | 5 (25) | 0.455 |
| Diuretics | 25 (18) | 6 (30) | 0.205 |
| Anti-diabetic | 37 (27) | 3 (15) | 0.263 |
| Statins | 97 (70) | 16 (80) | 0.331 |
| Anti-platelets | 88 (63) | 12 (60) | 0.775 |
| ***Molecular Profile*** |  |  |  |
| PCSK9, ng/mL | 245±83 | 288±102 | **0.047** |
| Total-C, mg/dL | 176±46 | 180±44 | 0.710 |
| LDL-C, mg/dL | 93±41 | 98±44 | 0.563 |
| HDL-C, mg/dL | 56±16 | 55±15 | 0.955 |
| Triglycerides, mg/dL | 139±78 | 183±194 | 0.264 |
| FPG, mg/dL | 108±28 | 106±22 | 0.590 |
| insulin, μIU/ | 13.1±13.9 | 10.3±9.9 | 0.282 |
| hs-CRP, mg/dL | 0.3±0.4 | 0.29±0.27 | 0.984 |
| Interleukin 6, pg/mL | 2.09±4.65 | 1.35±1.18 | 0.579 |
| MMP9, ng/mL | 88±87 | 124±87 | **0.015** |
| ICAM1, ng/mL | 203±83 | 224±89 | 0.333 |
| VCAM1, ng/mL | 614±167 | 614±182 | 0.888 |
| ***Coronary PVs*** |  |  |  |
| Total PV, mm^3^ | 731±678 | 727±589 | 0.979 |
| Dense Calcium PV, mm^3^ | 121±151 | 87±60 | 0.327 |
| Fibrous PV, mm^3^ | 252±243 | 235±178 | 0.768 |
| Fibrous-fatty PV, mm^3^ | 132±125 | 141±108 | 0.768 |
| Necrotic Core PV, mm^3^ | 195±177 | 222±193 | 0.523 |
| ***Annual change PVs*** |  |  |  |
| Total PV, mm^3^ | 13±17 | 11±13 | 0.638 |
| Dense Calcium PV, mm^3^ | 10±13 | 6±9 | 0.194 |
| Fibrous PV, mm^3^ | -1.96±14 | -10±24 | **0.034** |
| Fibrous-fatty PV, mm^3^ | -0.8±5 | -0.6±8.9 | 0.928 |
| Necrotic Core PV, mm^3^ | 4±8 | 11±17 | **0.004** |

**Table S7. List of genes significantly associated to PCSK9 mRNA expression showing a P value ≤0.001 at RNA sequencing.**

| **Gene** | **r** | **P value** |
| --- | --- | --- |
| *MMP9* | 0,680835636 | 6,49325E-61 |
| *MAP2K6* | 0,588215056 | 4,94471E-34 |
| *MAP2K3* | 0,510109703 | 1,9611E-43 |
| *TLR2* | 0,471664528 | 4,06503E-15 |
| *DDIT3* | 0,470487154 | 2,14801E-48 |
| *NCF1* | 0,461777467 | 9,20341E-43 |
| *IRF7* | 0,451303741 | 4,03291E-32 |
| *NCF4* | 0,44361473 | 3,6203E-44 |
| *LY96* | 0,438752074 | 8,92132E-38 |
| *MAPK10* | 0,400728278 | 4,96352E-17 |
| *NLRP3* | 0,399804859 | 3,75864E-18 |
| *CYP1A1* | 0,398607962 | 9,35853E-54 |
| *HSPA1A* | 0,38479043 | 1,47852E-39 |
| *PYCARD* | 0,376097256 | 4,24092E-22 |
| *MAPK3* | 0,357440069 | 2,57693E-41 |
| *CYBA* | 0,354561684 | 1,23526E-21 |
| *RXRA* | 0,34679082 | 1,84087E-46 |
| *IFNA16* | 0,346050933 | 9,63216E-10 |
| *MAPK14* | 0,340616799 | 4,69452E-11 |
| *APOB* | 0,338307443 | 2,04319E-08 |
| *HSPA6* | 0,337032881 | 3,46605E-40 |
| *VAV1* | 0,333325024 | 1,35725E-34 |
| *RXRG* | 0,3328795 | 9,40617E-08 |
| *IFNA8* | 0,330027703 | 4,23143E-10 |
| *NCF2* | 0,327997072 | 3,32869E-31 |
| *APOA1* | 0,327263574 | 9,61205E-36 |
| *AGER* | 0,314289537 | 2,2136E-36 |
| *IFNA1* | 0,31297292 | 2,1505E-10 |
| *CAMK2B* | 0,311207702 | 4,68949E-11 |
| *CASP9* | 0,306110804 | 9,75435E-39 |
| *IL18* | 0,301534072 | 5,39035E-30 |
| *SELE* | 0,296214362 | 1,13153E-09 |
| *CAMK2A* | 0,295201883 | 9,09456E-09 |
| *TNFSF10* | 0,29055451 | 3,15942E-24 |
| *IFNA21* | 0,289678077 | 1,102E-08 |
| *RHOA* | 0,288332258 | 3,2975E-29 |
| *TNFRSF1A* | 0,28180416 | 1,25972E-33 |
| *CALM2* | 0,276327659 | 4,30856E-19 |
| *MAPK13* | 0,269986612 | 3,50919E-06 |
| *HSPA1L* | 0,266402465 | 5,68572E-31 |
| *ABCG1* | 0,265213979 | 5,07175E-24 |
| *CALML3* | 0,262663273 | 7,47319E-06 |
| *IFNA10* | 0,262663273 | 7,47319E-06 |
| *IFNA4* | 0,262663273 | 7,47319E-06 |
| *STAT3* | 0,260754054 | 9,44242E-09 |
| *CALML5* | 0,251636223 | 3,31974E-09 |
| *IFNA2* | 0,24905746 | 5,27771E-05 |
| *CYP2C9* | 0,248932472 | 3,16504E-08 |
| *IFNA6* | 0,246642314 | 2,03093E-05 |
| *NFKBIA* | 0,238816074 | 6,49764E-22 |
| *IFNA13* | 0,235488522 | 4,17867E-07 |
| *PLCB2* | 0,223801087 | 7,81946E-37 |
| *MMP3* | 0,220863586 | 1,76439E-07 |
| *BAD* | 0,2159275 | 1,99683E-11 |
| *ARHGEF1* | 0,214026151 | 2,26576E-23 |
| *IL12B* | 0,207260771 | 0,000105623 |
| *SELP* | 0,20139767 | 1,28905E-28 |
| *CD14* | 0,1983748 | 5,39603E-20 |
| *IFNA17* | 0,195722895 | 5,83501E-09 |
| *MAPK12* | 0,195640299 | 6,66135E-16 |
| *MYD88* | 0,191788919 | 4,21723E-05 |
| *MAP2K7* | 0,188134536 | 4,2342E-26 |
| *ATF4* | 0,18678742 | 3,99477E-10 |
| *AKT2* | 0,186080101 | 1,44384E-16 |
| *RAC1* | 0,18437769 | 3,22294E-23 |
| *LBP* | 0,182135426 | 3,24529E-12 |
| *FOS* | 0,175435973 | 4,18015E-06 |
| *CAMK2G* | 0,169836257 | 2,55276E-09 |
| *RXRB* | 0,167646096 | 5,09749E-23 |
| *SOD2* | 0,167444474 | 1,64206E-06 |
| *BID* | 0,16726208 | 1,37655E-30 |
| *CYP2A7* | 0,166482555 | 0,000198181 |
| *PIK3CD* | 0,160990733 | 1,82106E-18 |
| *RELA* | 0,156384324 | 4,80227E-13 |
| *AKT1* | 0,149431039 | 3,2722E-29 |
| *PIK3R2.1* | 0,146088741 | 5,38015E-10 |
| *CDC42* | 0,145629869 | 8,93404E-18 |
| *PLCB4* | 0,144061266 | 5,3324E-07 |
| *CALM3* | 0,141792409 | 1,03133E-11 |
| *BCL2L1* | 0,138097046 | 1,07565E-20 |
| *APOA4* | 0,132940509 | 2,19522E-06 |
| *IKBKG* | 0,130406995 | 1,22418E-19 |
| *PPARG* | 0,128114016 | 2,36927E-14 |
| *IFNA14* | 0,126644445 | 6,45593E-09 |
| *IRAK1* | 0,126496618 | 5,48147E-07 |
| *CASP1* | 0,121900532 | 1,46857E-15 |
| *MIB2* | 0,108028969 | 3,41508E-15 |
| *VLDLR* | 0,102323035 | 3,38856E-16 |
| *CALML4* | 0,100830171 | 5,99264E-17 |
| *VCAM1* | 0,091757404 | 0,000554314 |
| *CCL3L1* | 0,08995354 | 7,94573E-13 |
| *CXCL3* | 0,088927944 | 1,58399E-06 |
| *BAX* | 0,064240619 | 1,08013E-07 |
| *POU2F3* | 0,063142907 | 3,21261E-16 |
| *HSPA1B* | 0,060072908 | 1,62058E-12 |
| *HSPA2* | 0,047016647 | 8,10891E-05 |
| *CXCL1* | 0,046110879 | 2,12718E-06 |
| *ICAM1* | 0,042303372 | 2,18008E-24 |
| *MMP1* | 0,016135946 | 5,67654E-10 |
| *IRF3* | 0,013676735 | 1,27673E-06 |
| *KRAS* | 0,010263152 | 0,0006476 |
| *TRAF6* | -0,013648451 | 7,61664E-05 |
| *PLCB1* | -0,014191356 | 3,35486E-07 |
| *TIRAP* | -0,018818392 | 0,000477283 |
| *TBK1* | -0,037145219 | 5,40571E-05 |
| *PLCB3* | -0,040425998 | 9,27602E-07 |
| *ROCK2* | -0,052970668 | 1,7538E-05 |
| *IL12A* | -0,055127202 | 3,51918E-10 |
| *PIK3CA* | -0,057182055 | 1,54385E-05 |
| *IL1B* | -0,063366823 | 1,47241E-08 |
| *CYP2J2* | -0,063400565 | 3,91699E-09 |
| *CALM1* | -0,069150566 | 7,65527E-16 |
| *JUN* | -0,072133292 | 4,46443E-11 |
| *MAPK8* | -0,078722643 | 3,06644E-07 |
| *PDPK1* | -0,080252828 | 0,000156764 |
| *NOS3* | -0,095400436 | 4,88827E-06 |
| *MIB1* | -0,095719774 | 4,05892E-07 |
| *RAP1B* | -0,100437801 | 2,96155E-09 |
| *CD40* | -0,107327566 | 2,20695E-06 |
| *LDLR* | -0,127127441 | 3,22107E-31 |
| *TICAM2* | -0,128544384 | 4,91716E-07 |
| *HSPA4* | -0,128725128 | 2,08313E-26 |
| *CD36* | -0,134678252 | 1,43658E-06 |
| *PTK2* | -0,141895723 | 1,61518E-11 |
| *FASLG* | -0,142246143 | 6,20491E-25 |
| *CYBB* | -0,144510375 | 4,03069E-08 |
| *CASP3* | -0,145873208 | 5,46627E-07 |
| *ITPR1* | -0,146154115 | 9,51475E-14 |
| *NRAS* | -0,147137483 | 1,25106E-14 |
| *HSP90AB1* | -0,153641604 | 0,000398216 |
| *NFKB1* | -0,155165142 | 6E-07 |
| *MAP3K7* | -0,158307781 | 7,25534E-10 |
| *CYCS* | -0,165235704 | 1,10914E-21 |
| *PPP3CB* | -0,16663283 | 5,98412E-12 |
| *TNF* | -0,172779661 | 1,4885E-14 |
| *PIK3R1* | -0,179646175 | 1,09531E-17 |
| *HSP90AA1* | -0,181886916 | 1,88158E-13 |
| *BCL2* | -0,187444437 | 3,16449E-30 |
| *TNFRSF10A* | -0,191024032 | 4,78239E-25 |
| *CASP7* | -0,191089384 | 6,74316E-30 |
| *AKT3* | -0,196726137 | 1,15807E-20 |
| *PRKCA* | -0,203551931 | 1,61954E-20 |
| *EIF2AK3* | -0,212610037 | 2,72877E-19 |
| *MAPK9* | -0,224170929 | 1,10547E-23 |
| *PIK3R3* | -0,225106655 | 1,26083E-27 |
| *CASP6* | -0,232485932 | 4,37467E-39 |
| *HSP90B1* | -0,23421565 | 2,57112E-31 |
| *CD40LG* | -0,238654209 | 3,69723E-39 |
| *CAMK2D* | -0,245462443 | 1,70626E-29 |
| *NFATC2* | -0,246219613 | 3,72104E-41 |
| *XBP1* | -0,250669551 | 2,8772E-36 |
| *PPP3CC* | -0,25606103 | 5,08619E-34 |
| *HSPA8* | -0,291141572 | 1,64951E-10 |
| *PLCG1* | -0,291626881 | 1,68791E-30 |
| *TRAF3* | -0,2955581 | 6,61705E-27 |
| *EIF2S1* | -0,296179991 | 1,85418E-43 |
| *HSPD1* | -0,318755483 | 1,38163E-36 |
